# Binding orientation of weakly associating membrane peripheral proteins via membrane paramagnetic relaxation enhancement NMR

**DOI:** 10.1038/s42004-026-02037-z

**Published:** 2026-04-24

**Authors:** Olivier Soubias, Frank Heinrich, Paul A. Randazzo, R. Andrew Byrd

**Affiliations:** 1 Center for Structural Biology, Center for Cancer Research, National Cancer Institute, National Institutes of Health, Frederick, MD USA; 2 Laboratory of Cellular and Molecular Biology, Center for Cancer Research, National Cancer Institute, National Institutes of Health, Bethesda, MD USA; 3 Department of Physics, Carnegie Mellon University, Pittsburgh, PA USA; 4 NIST Center for Neutron Research, Gaithersburg, MD USA

**Keywords:** Solution-state NMR, Solution-state NMR, Membrane proteins

## Abstract

Cell-membrane signaling and trafficking rely on proteins that associate with lipid bilayers through dynamic, low-affinity interactions. Defining how these proteins dock onto membrane surfaces is therefore essential for understanding their function. While Neutron Reflectometry (NR) combined with molecular dynamics (MD) simulations is frequently used, complementary approaches that do not require access to large-scale neutron facilities are needed. Here, we establish membrane Paramagnetic Relaxation Enhancement (mPRE) Nuclear Magnetic Resonance (NMR), combined with nanodiscs as membrane mimics and optimized acquisition strategies, as an accessible solution for extracting membrane–protein distance constraints even for weakly bound systems. Using the PI(4,5)P₂-binding ASAP1 Pleckstrin Homology (PH) domain as a model, we show that both conventional mPRE and a new dynamic-exchange mPRE (EX-mPRE) method reproduce the membrane orientation obtained by NR. In addition, we show that increasing PI(4,5)P₂ levels to mimic nanoscale membrane clustering broadened the orientational distribution of ASAP1 PH. EX-mPRE, which transfers PREs from transient bound states to the observable free state, further enables studies of temperature-sensitive or rapidly exchanging membrane interactions. Together, these results provide the formalism and establish mPRE and EX-mPRE NMR as a powerful alternative for resolving the membrane orientation of peripheral proteins and for probing how lipid composition affects their behavior.

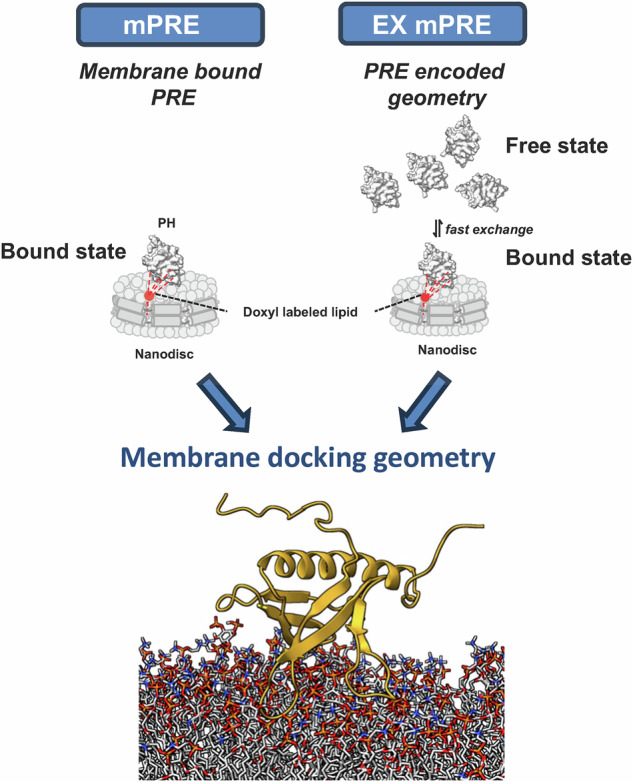

## Abstract (web summary)

Cell-membrane signaling depends on proteins that interact transiently with lipid bilayers, yet defining how they dock onto membranes has typically required neutron-based methods. Here, the authors combine membrane paramagnetic relaxation enhancement (mPRE) NMR with nanodiscs and optimized acquisition strategies to provide an accessible approach for determining membrane-protein orientation even in weakly bound systems.

## Introduction

Transient association between peripheral membrane proteins and various organelles membranes support essential cellular processes, including signaling, membrane trafficking, cytoskeletal assembly and dynamics, cell adhesion, and membrane homeostasis^1,2^. Although structural motifs that mediate membrane association, such as pleckstrin homology (PH) domains and other phosphoinositide- or curvature-sensing modules including C2, phox homology (PX), FYVE, and BAR domains have been identified, the precise binding geometries and atomic-level details of their interfaces with phospholipid bilayers remain largely unresolved^3–5^. This is despite the fact that a description of the interface, with its effects on both the membrane and protein structures, is necessary for a complete understanding of the critical biochemistry the proteins mediate. To date, neutron reflectometry (NR) has been the go-to method for determining the orientation of peripheral membrane proteins, often in combination with molecular dynamics (MD) simulations^6–9^. A range of other biophysical techniques has also been used to probe peripheral membrane protein and peptide orientation, including electron paramagnetic resonance (EPR) spectroscopy^10–12^, nuclear magnetic resonance (NMR) in solution^9,13–15^ or in the solid state^16–18^, small-angle neutron scattering (SANS)^19^, fluorescence spectroscopy^20–23^ and oriented circular dichroism^24,25^. However, EPR and fluorescence measurements typically require site-specific labeling; NMR approaches that rely on residual dipolar couplings (RDC)^26^ demand finding compatible orienting media, solid-state NMR requires substantial protein quantities; and access to both SANS and NR is severely limited, requiring approved experimental time at a large-scale neutron scattering facility. These limitations have motivated the search for alternative approaches capable of probing protein–membrane interactions in solution. Paramagnetic relaxation enhancement (PRE) NMR provides such an alternative and has been extensively used to characterize folded and unfolded proteins, complexes, and low-population states^27,28^. A membrane-focused variant, membrane-to-protein PRE (mPRE), uses bilayers doped with acyl-chain nitroxide-tagged lipids^9^ or lipid headgroup-chelated Gd³⁺ complexes^29,30^ to probe protein–membrane proximity in both stably bound peripheral proteins and integral membrane proteins^31,32^. Because mPRE NMR experiments are performed in solution, require only modest protein concentrations (e.g., 10–50 µM), and rely on straightforward sample preparation, they offer a particularly sensitive and accessible approach for mapping membrane‑interaction geometries. Importantly, unlike earlier PRE-based studies on *tethered* membrane proteins, such as small GTPases^9,13,33^, the proteins examined here belong to the class of *non-tethered, conditionally membrane-associated peripheral proteins* whose weak, reversible interactions with bilayers present a different biophysical challenge. Here, we examined two mPRE strategies designed to determine their membrane-binding orientation. The first relies on conditions in which the protein is predominantly membrane-bound, requiring high membrane concentrations, far above the membrane dissociation constant (*k*_d_), and elevated temperatures, but only two samples. The second method exploits dynamic exchange between membrane-bound and free states. Although it demands a larger set of samples, it allows data collection at lower temperatures, which is advantageous for studying heat-sensitive proteins. To maximize structural coverage, we combine amide backbone PREs and sidechain methyl group PREs. While methyl groups are often well distributed, methyl PREs depend strongly on side-chain placement and can leave gaps in surface sampling, particularly in small domains. We chose the ASAP1-PH domain, a 14 kDa protein which binds to phosphatidylinositol phosphate (PIP) PI(4,5)P_2_ and is implicated in Arf1·GTP hydrolysis, cell adhesion, and actin-driven cancer behavior^34,35^. The ASAP1 PH domain was chosen because its membrane orientation has recently been established through NR and MD simulations^8^. After optimizing the experimental conditions for both mPRE approaches, we measured PRE-derived pseudodistances between the membrane and nuclei throughout the backbone and methyl-bearing side chains to maximize structural constraints. We show that data from mPRE derived from both approaches define a docking geometry for ASAP1 PH consistent with prior reports, demonstrating that mPREs provide an effective alternative to NR. Furthermore, we found that a high PI(4,5)P_2_ concentration broadened the orientational distribution of ASAP1 PH at the membrane interface, supporting the idea that phosphoinositide concentration could play a role in regulating the orientation of PH domain-containing proteins, many of which are oncogenic. In summary, using the PH domain of ASAP1 as a model peripheral membrane protein, we were able to extend our understanding of ASAP1 and established that mPREs provide a broadly applicable, accessible, and robust alternative to NR for studying peripheral membrane proteins.

## Results and discussion

### NMR enhancements for backbone-based mPRE in nanodisc-bound proteins

**Fig. 1:**
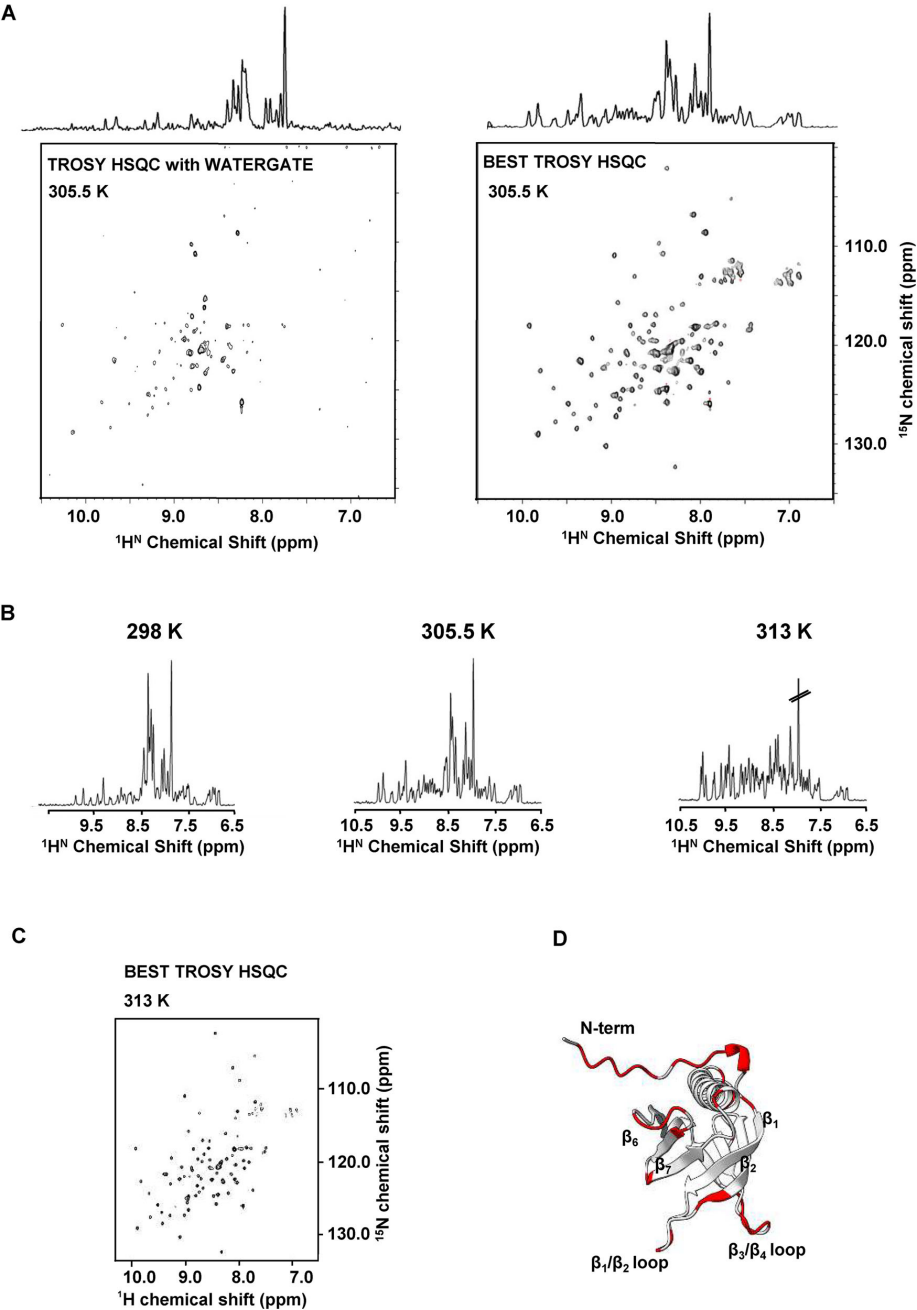
Optimized NMR conditions restore backbone amide signals of membrane bound PH domain. **A** ^1^H-^15^N HSQC obtained for membrane-bound U-^2^H, ^15^N ASAP1 PH using standard TROSY HSQC with WATERGATE (left), BEST-TROSY HSQC pulse sequence (right) at 305.5 K. **B** One-dimensional ^1^H NMR spectrum corresponding to the projection of a ^1^H-^15^N HSQC along the F1 dimension obtained for membrane-bound U-^2^H, ^15^N ASAP1 PH using a BEST-TROSY HSQC pulse sequence as a function of temperature. **C** Best TROSY spectrum of U-^2^H,^15^N ASAP1 PH domain bound to ND (PC:PI(4,5)P2, 95:5) at 313 K (50 μM, recycle delay = 5 s, 8 h acquisition time, [ND] 125 μM ~ 9 mM total lipids). **D** Structure of the ASAP1 PH domain. Observed residues are colored gray, residues that were not observed are red.

Our starting point was the standard mPRE framework, which assumes that the protein population exists almost entirely in the membrane-bound state. In this regime, the fraction of bound protein is very high (*χ*^*mbne*^ ~ 0.9–1) a condition we refer to as predominantly bound (PB) state. We previously reported that a methyl TROSY experiment^36^ enabled the observation of all methyl cross peaks of a U-^2^H, δ1-^13^C^1^H-labeled Isoleucine, δ1-^13^C^1^H-labeled Leucine, γ1-^13^C^1^H-labeled Valine and β-^13^C^1^H-labeled Alanine ASAP1 PH domain (Supplementary Fig. 1) at 298 K^8^, when bound to nanoscale lipid domains (nanodiscs, ND) formed using the belt protein MSPΔH5 (nanodisc molecular weight (MW) ~110 kDa)^37–39^. In contrast, at the same temperature, [^1^H–^15^N] TROSY NMR^40^ measurements using classical pulse sequences performed on a perdeuterated U-^2^H, U-^15^N-labeled PH domain at PB conditions produced spectra in which virtually all signals were absent, preventing backbone-membrane distance measurements. We hypothesized that the absence of amide cross peaks in the protein core stemmed from (1) amide proton (^1^H^N^) attenuation from water exchange and spin diffusion, caused by poor water suppression in the highly viscous media, and (2) rapid transverse relaxation due to slow molecular motion of the 100 kDa plus particle. While localized conformational exchange on the µs-ms timescale within the loops may contribute to line broadening, the global nature of the signal loss suggests that the combination of water exchange effects and slow molecular motion are the predominant factors. Therefore, we compared water-suppression pulse sequences (WATERGATE^41^, selective *π*^42^, and band-selective shaped pulses used in SOFAST-type experiments^43–45^) and examined the influence of raising the temperature on spectral quality, as originally demonstrated for ND-reconstituted OmpX^39^. In our hands, we found that employing band-selective shaped pulses for ^1^H^N^ excitation and raising the temperature to 313 K yielded the best results (Fig. 1A, B). Together, this improved the signal-to-noise ratio (*S*/*N*) by a factor of 42 compared to broadband ^1^H excitation with WATERGATE water suppression at 298 K. Simply raising the temperature from 298 to 313 K increased the *S*/*N* of backbone ^1^H^N^ by a factor of 2 (Fig. 1B) when band-selective shaped pulses for ^1^H^N^ excitation were employed. At 313 K using optimized experiments, 78 ^1^H^N^-^15^N cross peaks out of 126 residues could be measured, including most of the residues of the β-sandwich and C-terminal helix of the PH fold. Missing residues were in loop regions and within the unstructured N-terminal (325–339) and C-terminal (441–451) stretches of the PH domain^34^ (Fig. 1C, D). This is likely due to unfavorable dynamics and fast amide hydrogen exchange in flexible regions.

**Fig. 2:**
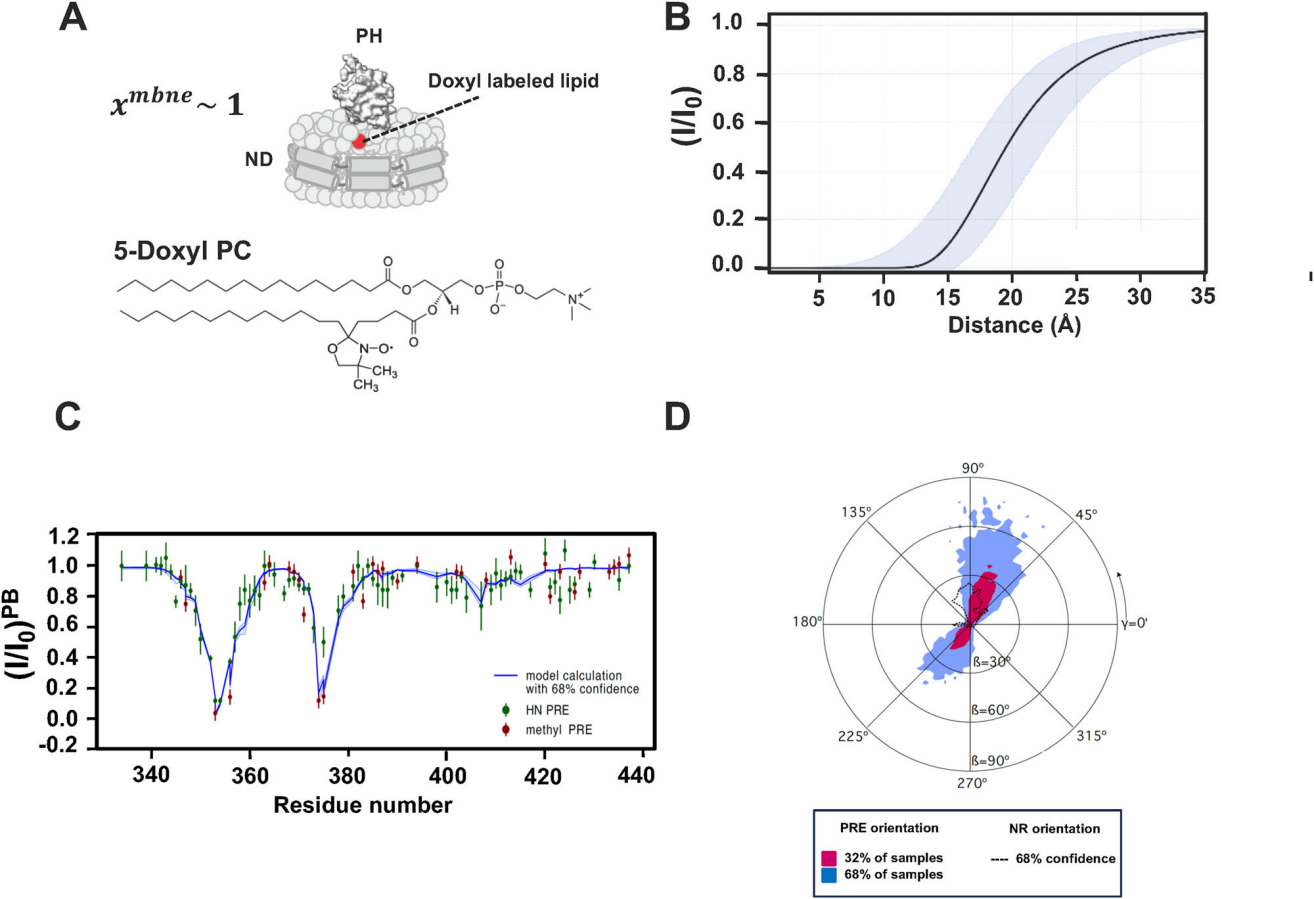
mPRE determination of ASAP1 PH orientation. **A** *Top*: cartoon of ASAP1 PH domain bound to nanoscale lipid domain (ND) containing a doxyl-labeled lipid. *Bottom*: chemical structure of 5-doxyl-16:0-16:0 PC used in this study. **B** Theoretical mPRE *I*/*I*_0_ ratio as a function of the distance *r* between the doxyl moiety and protein segment (black line) overlaid with the expected range of values when the doxyl position is treated as a Gaussian distribution within the bilayer (*σ* = ±3 Å; blue region). **C** H^N^ and H^CH3^ sidechain methyl PRE of ASAP1 PH measured at 5 mol% PI(4,5)P_2_ in the bilayer (3 mol% doxyl-5 PC). Experiments were performed at 50 μM U-^2^H,^15^N and δ1-^13^C^1^H-labeled isoleucine, δ1-^13^C^1^H-labeled leucine and γ1-^13^C^1^H-labeled valine, β-^13^C^1^H-labeled alanine ASAP1 PH, 120 μM ND, 313 K using a BEST TROSY pulse (recycle delay 5 s). Recycle delays exceeding 4 s were required to ensure accurate cross-peak intensity measurements, eliminating artifacts caused by differences in longitudinal *R*_1_ relaxation rates between spin-labeled and unlabeled samples. Data are presented as mean values. Error bars were calculated based on the signal-to-noise (*S*/*N*) ratio of the spectra as described in “Methods”. Model calculations and 68% confidence limits are based on rigid-body rotations and translations of the ASAP1 PH crystal structure with respect to a lipid bilayer, followed by back-calculations of the PRE signal. **D** Samples drawn from the fitted distributions of the two Euler angles *β* and *ɣ* that define the orientation of ASAP1 PH relative to the bilayer for at 5 mol% PI(4,5)P_2_ in the ND bilayer. Red and blue areas contain 32% and 68% of all sampled orientations, respectively. The dotted line indicates the 68% confidence interval for the single best-fit orientation of ASAP1 PH, as previously determined by neutron reflectometry^8^. For each fit iteration, PREs were calculated for a sample of 600 orientations and bilayer distances drawn from the parameterized multivariate normal distribution. The shortest distances between residues and the bilayer were used, thereby forgoing a sampling of possible positions of paramagnetic labels on the nanodisc due to the presence of more than one label in the lipid bilayer.

To record mPRE between the membrane and the protein, negatively charged ND composed of (14:0-14:0-PC) and (18:1-18:1-PI(4,5)P_2_) lipids containing 5 mol% PI(4,5)P_2_ (equivalent to two PI(4,5)P_2_ per PH domain) were used as a diamagnetic reference. Paramagnetically tagged ND were obtained by adding 3 mol% nitroxide spin-labeled 1-palmitoyl-2-stearoyl-(5-doxyl)-sn-glycero-3-phosphocholine (5-doxyl PC) while keeping the PC: PI(4,5)P_2_ ratio constant (Fig. 2A). We chose 5-doxyl PC instead of headgroup-chelated probes, such as Gd^3+^-headgroup chelated lipids^13^ to maintain the native electrostatics of the ND bilayer interface. Locating the nitroxide within the upper part of the acyl chain (position 5-versus 10- or 14-doxyl) avoids perturbing the charged lipid environment required for conditional peripheral membrane protein binding, yet remains close enough to capture their membrane interactions effectively. In addition, while headgroup chelated labels may show stochastic partitioning (e.g., tempo-PC) and concentration-dependent depth variability^46^, the 5-doxyl group remains fixed at the depth dictated by the acyl-chain geometry, with essentially no deviation from the corresponding unlabeled carbon even at high labeling spin-labeled lipid concentration in the bilayer^47–49^.

The molar fraction of 5-doxyl PC was chosen to have, on average, one spin-labeled lipid per lipid monolayer. The ND concentration was tightly controlled to ensure a variation of less than 1% between diamagnetic and paramagnetic samples. This ensured that changes in relaxation rates were solely attributable to the spin-labeled lipid. Initial ND concentrations were estimated via UV–visible spectrophotometry based on the absorbance of MSPΔH5 at 280 nm. However, to ensure the high precision required for binding isotherms, particularly given that small variations in PI(4,5)P_2_ can significantly alter the effective binding affinity, we found that the most reliable way to determine ND concentrations was to monitor ¹H–¹³C chemical shift perturbations (CSPs) of methyl residues in ASAP1 PH that report on binding yet remain sufficiently distant from the nitroxide to be quantifiable (residues I371, A381, L383, and L402)^8^. These CSPs, measured within the linear region of the binding isotherm, were then compared between NDs with and without spin label to determine the exact ND concentration.

As it was shown earlier^50^, the PRE is proportional to <*r*^−6^> (where *r* is the distance between the nucleus of interest and the unpaired electron). Extensive MD simulations and EPR studies have established that the 5-doxyl group maintains a stable, Gaussian distribution centered at approximately 8 ± 3 Å below the bilayer phosphate plane^46^ and, because of the large magnetic moment of the unpaired electron, the PRE effect is detectable for nucleus-electron distances extending up to 25 Å for a nitroxide spin-label^51–53^ (Fig. 2B). Distance restraints were derived from residue-specific intensity ratios ((*I*/*I*₀)^PB^) measured with and without spin-labeled lipids. As shown in Fig. 2C, (*I*/*I*₀)^PB^ values from backbone amide resonances (H^N^-PRE) and methyl probes (H^CH₃^-PRE) are in good agreement. Significant (*I*/*I*₀)^PB^ attenuation was observed for residues within residues in the *β*_1_/*β*_2_ (residues 350–357) and *β*_3_/*β*_4_ loops (residues 372–380), indicating proximity of those residues to the doxyl group embedded in the hydrophobic core of the bilayer. By applying a distance-dependent model, we can map semi quantitatively these PRE profiles to an immersion depth. For example, the (*I*/*I*₀)^PB^ of ~0.4 measured for residues K350 and W357 corresponds to an average distance of ~18 Å from the spin label and place the tip of the *β*_1_/*β*_2_ loop approximately 3 Å beneath the phosphate headgroup plane, in good agreement with previous results. In addition, low (*I*/*I*₀)^PB^ is visible on the H^N^ PRE profile for residues 400–410 and is missing in the corresponding H^CH3^ PRE profile, underscoring the value of measuring backbone constraints.

To determine the orientation of ASAP1 PH at the membrane, we back-calculated NMR-PRE data from ensembles of ASAP1 PH orientations and distances from a (virtual) lipid bilayer membrane using a previously published method^7^. Briefly, ensembles were created by rigid body rotations of ASAP1 PH from a reference orientation using two Euler angles *β* and *ɣ* (see ref. ^8^ for details). Thereby, both angles were sampled from a 2-dimensional multivariate normal (MVN) distribution centered around a central orientation (*β*_0_, *ɣ*_0_) with standard deviations *σ*_β_ and *σ*_ɣ_, and a correlation ρ between the two angles. The distance of ASAP1 PH from the membrane modeled separately as a normal distribution centered on a central distance (*d*) with standard deviation (*σ*_d_). All seven parameters were simultaneously optimized using a Monte Carlo Markov chain-based global optimizer, from which we report median fit parameter results and 68% confidence limits^9^. The fit results are presented in Fig. 2D and Supplementary Table 1. The MVN model yields an excellent agreement with the PRE data, with goodness-of-fit (normalized *χ*²) values of 1.9. The data show that the ASAP1 PH domain binds in an upright orientation, with the β-barrel axis aligned to the bilayer normal and the *β*_1_/*β*_2_ and *β*_3_/*β*_4_ loops at the membrane interface, rocking within a narrow range of orientations along the long axis of the C-term α-helix during which the loops remain in contact with the lipid membrane. This result closely matches earlier NR findings (Fig. 2D)^8^. The analysis also provides new insight: NR was limited to modeling a single average orientation, whereas mPRE captures a distribution of ASAP1 PH domain orientations using the MVN model.

### EX-mPRE: capturing the geometry of transiently bound proteins at moderate temperatures

**Fig. 3:**
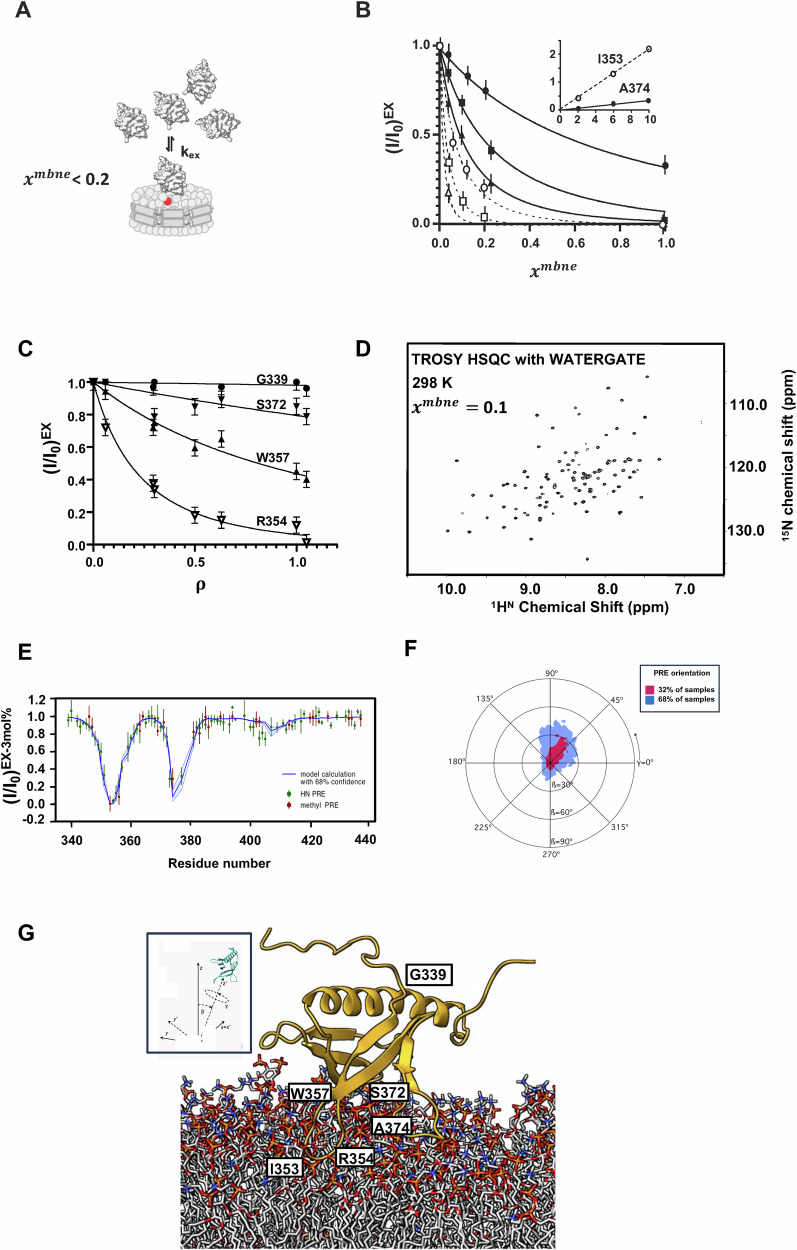
EX mPRE determination of ASAP1 PH orientation. **A** Cartoon of the ASAP1 PH domain in exchange with ND. **B** TROSY HSQC spectrum of U-^2^H,^15^N ASAP1 PH domain bound to ND (PC:PI(4,5)P2, 95:5) at 298 K (50 μM, recycle delay = 5 s, 1.5 h acquisition time, [ND] 8 μM ~ 0.5 mM total lipids). **C** NMR-PRE ratios (*I*/*I*_0_)^EX^ for ASAP1-PH A374-[^13^CH_3_]^β^ (filled symbols) and I353-[^13^CH_3_]^δ^ (open symbols) as a function of bound ASAP1-PH to PC:PIP(4,5)P2 NDs containing 2 (circle), 6 (square), and 10 (triangle) mol% of doxylPC-5. Data are presented as mean values. Error bars were calculated based on the signal-to-noise (*S*/*N*) ratio of the spectra as described in “Methods”. A value for *Γ*_2_*p* is obtained for each 5-doxyl PC concentration using Eq. 2. ${\,\varGamma }_{2}^{{free}}$ was estimated from the half-height line width of peaks in the diamagnetic spectra. *Inset*: linear relationship between ^1^H-*Γ*_2_*p* and bilayer concentration of doxylPC-5. Concentration of ASAP1- PH was 50 μM. The fraction of bound ASAP1-PH was determined by measuring chemical shift changes of I371-[^13^CH_3_]^δ^, A381-[^13^CH_3_]^β^, and L383-[^13^CH_3_]^proS^ methyls as a function of ND concentration and compared to data reported in (**D**). Fit of the data to Eq. 3 for glycine 339 (G339, closed circle), serine 372 (S372, inverted closed triangle), tryptophan 357 (W357, closed triangle), and arginine 354 (R354, inverted open triangle) **E** H^N^ and H^CH3^ sidechain methyl PRE of ASAP1 PH calculated at 3 mol% of 5-doxyl PC from the fitted ${\,\varGamma }_{2}^{{mbne}}$ (at 5 mol% PI(4,5)P_2_). **F** Same as Fig. 2D but calculated from data acquired with the exchange membrane PRE strategy. **G** *Left*: protein orientations are realized by first rotating the reference orientation of the high-resolution structure by an angle *β* about the *x*. Second, the structure is rotated by an angle *γ* about *z*′. *Right*: visualization of the PH domain structure in a configuration (orientation and insertion depth) on the bilayer at the center of the 68.2% probability region shown in Figs. 2D and 3F. Data are presented as mean values. Error bars were calculated based on the signal-to-noise (*S*/*N*) ratio of the spectra as described in “Methods”.

The requirement for prolonged data acquisition at temperatures of 313 K or higher can be a limitation for some proteins, making lower temperatures key for protein stability. In addition, mPRE measured under predominantly bound (PB) conditions is constrained by the MW of the entire membrane-bound complex, which can limit applicability for assemblies larger than 150 kDa. We introduce a second approach, termed EX-mPRE, which does not require elevated temperatures, that relies on PRE’s ability to reveal structural information about transient, low-population states within exchanging systems (EX conditions, Fig. 3A). As shown earlier^51^ and references therein, if two states exchange with one another, the PREs from the minor state can be encoded on the PREs measured on the observable major state providing that (1) the paramagnetic center-proton distances in the minor state is shorter than in the major state, and (2) the exchange rate (*k*_ex_) between the two species is fast enough to permit the transfer of PREs from the minor state to the observable major state.

In a previous study, we determined that ASAP1-PH domain binds to membrane containing 5 mol% of PI(4,5)P_2_ with a *K*_d_ of 12 ± 3 μM. Assuming a diffusion-limited rate of binding (*k*_on_), the resulting dissociation rate *k*_off_ of the protein-membrane complex is estimated to be between 10,000 and 20,000 s^−1^. If the exchange is fast on the PRE time scale, then the observed PRE measured on the major species will be a population-weighted average of the transverse relaxation rates $\left({\varGamma }_{2}\right)$ in the two states (Eq. 1).

1 $${\varGamma }_{2}^{{app}}={{\chi }^{{free}}\cdot \varGamma }_{2}^{{free}}+{{\chi }^{{mbne}}\cdot \varGamma }_{2}^{{mbne}}$$

with ${\chi }^{{mbne}}$ $\left({\,\varGamma }_{2}^{{mbne}}\right)$ and ${\chi }^{{free}}$ $\left({\,\varGamma }_{2}^{{free}}\right)$ the fraction of protein (the relaxation rate) in the free and membrane-associated state, respectively.

We used NDs prepared with the same lipid composition as before. A total of 7 samples were analyzed (Supplementary Table 2) at a low fraction of bound protein (*χ*^*mbne*^ < 0.2) and 5-doxylPC mole fraction $\left({\chi }^{{dox}}\right)$ of 2, 6, and 10 mol%, while keeping the PC:PI(4,5)P_2_ ratio constant. Fig. 3B plots the ratios *I*/*I*_0_ (noted *I*/*I*_0_^EX^) measured for ASAP1- PH A374-[^13^CH_3_]^β^ and I353-[^13^CH_3_]^δ^ as a function of ${\chi }^{{mbne}}$ in the presence of NDs containing increasing concentration of paramagnetically tagged lipid and analyzed according to Eq. 2, modified from ref. ^53^.

2 $${\left(\frac{I}{{I}_{0}}\right)}^{{\mathrm{EX}}}=\frac{{\,\varGamma }_{2}^{{free}}\,\cdot {e}^{-\left({\chi }^{{mbne}}\cdot {\,\varGamma }_{2}^{{mbne}}\cdot \,t\right)}}{{\,\varGamma }_{2}^{{free}}+{\chi }^{{mbne}}\cdot {\,\varGamma }_{2}^{{mbne}}}$$

where *t* is the total evolution time in the HMQC experiment. As expected, *I*/*I*_0_^EX^ decays as ${\chi }^{{mbne}}$ increases. The decay was well fitted with ^1^H-*Γ*_2_ values of 51 ± 10, 146 ± 20, and 292 ± 40 s^−1^ for A374-[^13^CH_3_]^β^ and 444 ± 92, 1048 ± 102, and 2204 ± 200 s^−1^ for I353-[^13^CH_3_]^δ^ at 2, 6, and 10 mol% of 5-doxylPC, respectively. The fact that these decays remain well-fitted by a single exponential even at the highest PRE of ~2200 s^−1^ observed for I353 confirms that the rate of exchange between bound and free ASAP1-PH remains significantly faster than the ^1^H-*Γ*_2_ relaxation rate. This ensures that the measured PREs represent a true population-weighted average.

Interestingly, ^1^H-*Γ*_2_ values increased linearly with the concentration of 5-doxylPC (Fig. 3B, Inset). This indicates that the mPRE effect can be seen as a single nucleus-electron spin process under the conditions of the experiment. In other words, at any given time, the distance between a particular proton and a second spin label is large enough such that ^1^H-*Γ*_2_ of that particular proton only depends on the probability to be close to one spin label, when the spin label concentration is kept below 10 mol%. As a result, all *I*/*I*_0_^EX^ data, measured across varying bound fractions and 5-doxylPC mole fractions, were subsequently aggregated and analyzed according to Eq. 3 (Fig. 3C).

3 $${\left(\frac{I}{{I}_{0}}\right)}^{{\mathrm{EX}}}=\frac{{\,\varGamma }_{2}^{{free}}\,\cdot {e}^{-\left(\rho \cdot {\,\varGamma }_{2}^{{mbne}}\cdot \,t\right)}}{{\,\varGamma }_{2}^{{free}}+\rho \cdot {\,\varGamma }_{2}^{{mbne}}}$$

where $$\rho ={\chi }^{{mbne}}\cdot \frac{{x}^{{dox}}}{2}$$.

Since the dominant species is now the 14 kDa PH domain in solution, water suppression is more robust. This allowed us to record [^1^H–^15^N] HSQC NMR spectra at ambient temperature without the need of band-selective pulses (Fig. 3D). It also benefits from the narrow linewidths of the free state, improving distance precision and eliminating the need to assign resonances of the membrane-bound form (Supplementary Fig. 2). Out of 126 residues, 84 H^N^ cross peaks could be measured. Fig. 3E combines *I*/*I*_0_^EX^ for side chain methyls and H^N^ protons calculated at 3 mol% of 5-doxylPC in the bilayer. A comparison of Figs. 2D and 3F shows remarkable agreement between mPRE measured at PB and at EX conditions, resulting in a docking geometry of ASAP1 PH that is consistent between the two approaches and with previous NR data (Supplementary Table 3). It is advantageous to perform membrane PRE experiments as a function of the concentration of tagged lipid ${\chi }^{{dox}}$ and bound fraction ${\chi }^{{mbne}}$. For instance, at *χ*^*mbne*^ = 1 and 5 mol% of 5-doxylPC as in ref. ^8^ or at 3 mol% as herein, I/I_0_ measured for I353 and A374 are identical and equal to zero, corresponding to ^1^H-*Γ*_2_ ≥ 40 s^−1^. From those experiments, one can only determine that I353 and A374 sidechain methyls are within 16 Å of the doxyl-tagged lipid (see Fig. 2A). The experiments at increasing ${\chi }^{{mbne}}$ yield a much larger ^1^H-*Γ*_2_ for I353 than for A374. This indicates that I353 inserts deeper into the ND bilayer than A374, in agreement with previously published MD simulation results^8^. In a membrane PRE experiment, concentration- dependent experiments are therefore equivalent to having a paramagnetic tag with a different isotropic g-factor, improving the precision of the docking geometry^51,52^.

### Influence of PIP2 bilayer concentration on ASAP1 PH docking geometry

**Fig. 4:**
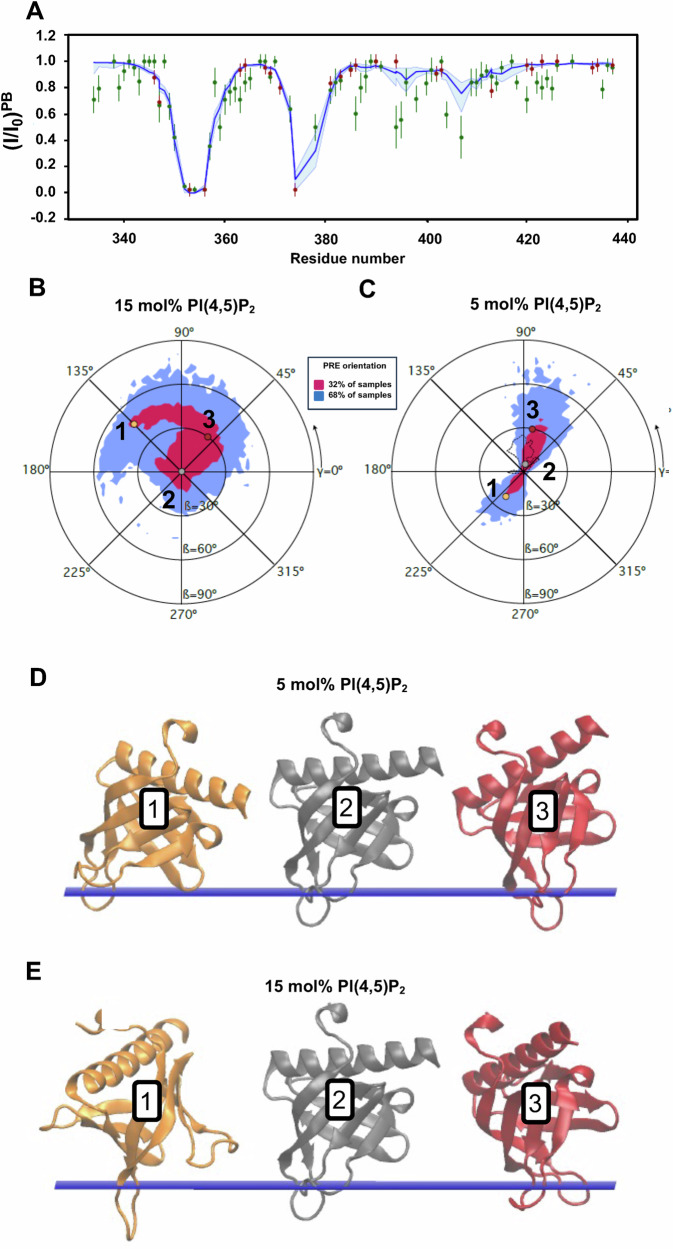
Influence of PIP2 bilayer concentration on ASAP1 PH membrane orientation. **A** H^N^ and H^CH3^ sidechain methyl PRE of ASAP1 PH measured at 15 mol% PI(4,5)P_2_ in the ND bilayer (3 mol% doxyl-5 PC). Data are presented as mean values. Error bars were calculated based on the signal-to-noise (*S*/*N*) ratio of the spectra as described in “Methods”. **B**, **C** Samples drawn from the fitted distributions of the two Euler angles *β* and *ɣ* that define the orientation of ASAP1 PH relative to the bilayer at 5 mol% and 15 mol% PI(4,5)P_2_ in the bilayer. Red and blue areas contain 32% and 68% of all simulated orientations, respectively. **D** Exemplary orientations of ASAP1 PH along the 32% confidence ridge for distributions of the protein orientation at lipid bilayers containing 5 mol% PI(4,5)P_2_. From left to right: (*β*, *ɣ*) = (20°, 235°), (5°, 70°), and (30°, 70°). The blue plane indicates the lipid headgroup-water interface. ASAP1 PH adopts a rocking motion along a trajectory that maintains contact between the *β*_1_/*β*_2_ and *β*_3_/*β*_4_ loops and the lipid bilayer. **E** Exemplary orientations of ASAP1 PH along the 32% confidence ridge for distributions of the protein orientation at lipid bilayers containing 15 mol% PI(4,5)P_2_. When increasing the PI(4,5)P_2_ concentration to 15 mol%, orientations in which one of the loops loses contact with the bilayer become possible.

mPRE experiments combined with nanodiscs, which accommodate a far broader range of lipid compositions than the tethered single-bilayer systems used for NR, can be used to address questions central to peripheral-membrane-protein function. For instance, despite low PIP concentrations in the cellular membranes (<5% in the plasma membrane, ~10% in Golgi membranes^54,55^), their ability to form nanoscale clusters is thought to enhance protein interactions^56,57^. Recently, a MD simulation study suggested PIP clustering can control the orientation of a PH domain in a concentration-dependent manner^58^. To probe the effect of high PIP concentration on ASAP1 PH orientation experimentally, we measured mPRE at 15 mol% of PI(4,5)P_2_ in the ND bilayer i.e., ~6 PIP2 per PH domain (Fig. 4A). All measurements were carried out under PB conditions, since increasing phosphoinositide concentration strengthens PH-domain binding (apparent *K*_d_ < 1 µM)^8,59^ and slows exchange beyond the regime of the EX-mPRE technique (Supplementary Fig. 3). Under these conditions, the effective binding stoichiometry is inherently difficult to determine because high PIP₂ density and low nanodisc concentration increase the likelihood that multiple PH domains occupy a single ND, making the interpretation of an EX-PRE experiment difficult. To ensure predominantly single‑occupancy binding under these conditions, we subsequently used a large excess of nanodiscs so that, stochastically, each nanodisc is most likely to host only one PH domain. Fig. 4B shows that increasing PI(4,5)P_2_ to 15 mol% significantly broadened ASAP1 PH orientations, while maintaining the center position of the distribution roughly the same as at 5 mol% (Fig. 4C and Supplementary Table 4). The data suggests that the interaction of ASAP1 PH with the bilayer surface is sensitive to the concentration and, potentially, the nanoscale clustering of PI(4,5)P_2_. At 5 mol% PI(4,5)P_2_ in the bilayer, the data reveal an anisotropic free energy landscape for ASAP1 PH, allowing for a rocking motion of the protein along the long axis of the C-term α-helix during which the two *β*_1_/*β*_2_ and *β*_3_/*β*_4_ loops remain in contact with the lipid bilayer (Fig. 4D). Increasing the PI(4,5)P_2_ concentration to 15 mol% leads to increased rolling motions of the C-terminal α-helix. Consequently, in some configurations, only one of the loops could maintain bilayer insertion (Fig. 4E). While our fitting procedure relies on a static PDB structure and it does not explicitly account for methyl-side-chain dynamics, our results are consistent with MD simulations of the general receptor for phosphoinositide 1 (GRP1) PH which reveal membrane associated poses where only one loop or binding site remains inserted, while others disengage as the protein samples different orientations as a function of PIP availability^58^ (Fig. 4B (gold circle) and 4E). Here, it suggests that contacts between the positively charged sidechain and the ND bilayer interface, rendered possible by the higher density of negatively charged PI(4,5)P_2_ headgroups at the interface, broadens the free energy landscape of possible orientations of the ASAP1 PH domain. In addition to the increased opportunities for specific contacts between basic sidechains and PI(4,5)P₂ headgroups, more general electrostatic effects may also contribute to the broadened orientational distribution observed at higher PIP densities. An increase in overall negative surface charge can enhance nonspecific attraction of the positively charged PH domain surface, thereby reducing the energetic penalty for sampling orientations that deviate from the canonical binding geometry, as seen with the addition of lipids with phosphatidylserine headgroups^8^. In this sense, the widened distribution may reflect not only enhanced dynamic sampling but also a partial relaxation of strict orientational constraints imposed by specific lipid–protein interactions. Because membrane binding domains (MBD) are frequently embedded within larger multidomain proteins, this dynamic mode of membrane association is likely to influence the regulation of MBD-containing proteins, many of which are oncogenic^60,61^. While our current study focuses on the isolated PH domain, the mPRE approach is well-suited to probe how lipid composition shapes the orientation of full-length, multidomain proteins and how partner binding further constrains or reorganizes these membrane-associated states. Future applications of mPRE to complexes or larger constructs will help clarify the extent to which lipid-driven orientational plasticity is preserved or overridden by protein–protein interactions.

## Conclusion and perspectives

Although mPRE had been previously used for mapping protein–membrane proximity, its application to *low-affinity, non-tethered* systems required the development of dedicated NMR strategies. By optimizing water suppression through band-selective excitation, acquiring data at elevated temperatures and the use of nanodiscs, we enabled reliable detection of backbone amide signals under predominantly bound conditions, allowing extraction of robust backbone-derived PRE restraints for a protein-bilayer complex of ~100 kDa, with an upper limit set by the overall tumbling of the *complex* and therefore consistent with the typical ~150 kDa limit of solution-state NMR for globular assemblies. Complementing this, the dynamic-exchange mPRE (EX-mPRE) approach described here provides an elegant solution for systems that cannot tolerate high temperatures. By transferring PREs from a transient, low-population bound state to an observable free state, EX-mPRE parallels other exchange-based NMR methods, such as chemical exchange saturation transfer^62,63^, dark state exchange saturation transfer (DEST)^64^, relaxation dispersion^65,66^ or transferred NOESY^67^, enabling detection of minor membrane-interacting states. This makes EX-mPRE particularly powerful for weakly binding peripheral domains, with membrane affinities in the low tens of micromolar to millimolar range, disordered regions that sample membrane contacts as elegantly shown in ref. ^68^, or small ligands that transiently partition into bilayers. Because EX-mPRE observes the *free* state, its molecular-weight limit is mostly governed by the size of the protein itself rather than the size of the membrane-bound complex. As a result, EX-mPRE retains the typical ~150 kDa limit for amide-based detection, independent of the size of the nanodisc to which the protein binds. Moreover, if the analysis is restricted to side-chain methyl probes, the accessible size range increases substantially, as methyl-TROSY detection remains effective for complexes in the 300–500 kDa regime and, in favorable cases, possibly even larger assemblies. Finally, another advantage of the approach is the ability to tune PRE strength through controlled variation of both the fraction bound and the concentration of spin-labeled lipids further enhances depth and orientation resolution.

A broader conclusion emerging from this work is that while mPRE can function as a stand-alone approach, as shown here, the authors believe that a multi-method strategy is essential for defining the structural and dynamic features of weakly associating peripheral membrane protein interactions. The measurements of RDCs, for example, would provide orientational constraints that directly report on tilt and roll angles, complementing the distance-based information obtained from mPRE as elegantly shown in ref. ^14^. Although RDC measurements remain challenging for highly charged peripheral membrane proteins due to incompatibility with many alignment media, the development of magnetically orientable nanodiscs could offer a promising route for collecting RDCs under “native” binding conditions. mPRE data also integrates naturally with MD simulations. Unbiased MD often suffers from slow rotational convergence and force-field sensitivity, particularly for peripheral membrane proteins whose membrane interactions are shallow and dynamic. PRE-derived $< {r}^{-6} >$ restraints provide information that is difficult to sample accurately in silico despite recent progress^69^, thereby validating MD-derived orientation ensembles. Beyond MD and RDCs, mPRE is synergistic with scattering methods, such as SANS or NR. Together, they can define how peripheral proteins orient and engage the bilayer, and how they contribute to the assembly of membrane‑active complexes, with far greater precision than any single method alone.

## Methods

### Preparation of the ASAP1 PH domain

The sequence for mouse ASAP1 PH domain, [325–451]-ASAP1, was cloned between Nde I and Bam HI restriction sites of the pET3a vector, which was then transformed into *Escherichia coli* BL21 Star (DE3) cells (Invitrogen) for protein expression, plated on LB agar containing carbenicillin (100 mg/L), and incubated overnight (o/n). For the production of [U-^2^H], [U-^15^N]-methyl specifically labeled protein, NH_4_Cl is substituted by ammonium chloride (^15^N ≥ 99%, Sigma-Aldrich, 299251), d-glucose is replaced by d-(^2^H, ^12^C)-glucose (^2^H ≥ 98%, Cambridge Isotope Laboratories, Inc; DLM-2062-10), and ^13^CH_3_-methyl specifically labeled precursors are added as described below. For a typical cell culture of 500 ml, a few freshly transformed colonies of BL21 (DE3) cells were picked to inoculate 5 ml of M9/H_2_O minimal media for o/n growth at 37 °C in a shaking incubator (250 rpm). One microliter of the o/n culture [typical optical density at 600 nm (OD_600_) ~ 1.2] was then used to inoculate 4 ml of fresh M9/H_2_O medium to achieve a starting OD_600_ of 0.25. At OD_600_ ~ 0.5, 5 ml of M9/D_2_O minimal media was added, and cell growth continued until an OD_600_ of ~0.5 is reached. Cells were diluted again by a factor of 2, and growth followed to OD_600_ ~ 0.5. This cycle was repeated until a D_2_O/H_2_O ratio of 3:1 (20 ml total) is reached. Cells were then harvested by centrifugation (3000 × *g* for 30 min) and resuspended in 25 ml of M9/D_2_O, and growth was continued in a 100-ml baffled flask until an OD_600_ of 0.5 is reached, before an additional 25 ml of M9/D_2_O was added for o/n growth at 37 °C. When the o/n OD_600_ was between 1.3 and 1.5, the o/n cell expression (50 ml) was added to 500 ml of M9/D_2_O, and growth followed at 37 °C, up to OD_600_ ~ 0.6. For selective I-[^13^CH_3_]^δ^, L-[^13^CH_3_]^proS^, V-[^13^CH_3_]^proS^ and A-[^13^CH_3_]^β^ methyl labeling, the QLAM-A_β_I_δ1_LV_proS_T_γ_ kit was used (NMR-Bio). After the addition of the precursor according to the manufacturer’s protocol, cell growth continued until an OD_600_ of approximately 0.8 at 20 °C is reached, at which time protein expression was induced with the addition of 1 mmol/L (mM) isopropyl β-d-1-thiogalactopyranoside (IPTG, Goldbio, I248C50). After induction, another 2 g/liter of d-(^2^H, ^12^C)-glucose was added, and the culture was grown o/n at 20 °C.

ASAP1 PH was purified by resuspending in buffer A (50 mM Tris pH 7.4, 150 mM NaCl) by using 30 mL buffer for 1 liter’s worth of cell pellet. A single protease inhibitor tablet (cOmplete EDTA-free, Roche) was used for each cell pellet. The cells were lysed using a cell disruptor (Microfluidics) and then ultracentrifuged (48,000 *× g* for 30 min) at 4 °C. Afterward, the supernatant containing the PH domain was removed, and all subsequent purification steps were conducted at room temperature, as it was observed that the PH domain precipitates when chilled. The supernatant was applied to a 5 mL HiTrap SP HP column (Millipore Sigma) pre-equilibrated with buffer A, washed with 10 column volumes (CVs) of buffer A, then eluted with a 6 CV linear gradient from buffer A to buffer B (50 mM Tris, pH 7.4, 1 M NaCl). Eluates containing PH domain were pooled and then injected onto a ~ 120 mL HiPrep 16/60 Sephacryl S-100 HR SEC column (Millipore Sigma) pre-equilibrated with buffer A supplemented with 1 mM TCEP. SEC eluates containing PH were then pooled, concentrated using 3000 MWCO spin-concentrators (Amicon), and snap-frozen using liquid nitrogen. Concentration of the PH domain was determined by ultraviolet (UV) spectroscopy (*ε*^280^ = 16,960 M − 1 cm^−1^).

### Preparation of nanodiscs for NMR spectroscopy

The plasmid for MSPΔH5 was a gift from F. Hagn and G. Wagner (Harvard Medical School). The protein was expressed and purified as described previously^39^. All lipids were purchased from Avanti Polar Lipids, Inc. To prepare nanodiscs, acyl chain perdeuterated 1,2-dimyristoyl-sn-glycero-3-phosphadidylcholine (DMPCd54) in chloroform solution (Sigma-Aldrich 319988), 1,2-dioleoyl-*sn*-glycero-3-phospho-(1′-myo-inositol-4′,5′-bisphosphate) (PI(4,5)P_2_) in chloroform: methanol (Millipore MX0488): water (20:9:1) solution were mixed and air-dried with nitrogen flow before solubilization with cholate in aqueous buffer [20 mM tris-HCl (pH 7.4), 150 mM NaCl, and 75 mM sodium cholate (Millipore SX0420)]. For paramagnetic samples, 1-palmitoyl-2-stearoyl-(5-doxyl)-sn-glycero-3-phosphocholine (5-doxyl PC) were incorporated, replacing a small fraction of DMPCd54. Nanodiscs were assembled by mixing MSPΔH5 with solubilized lipids at a ratio of 1:45 (final cholate concentration of 18 mM), followed by the removal of cholate from the mixture with Bio-Beads SM2 resin (Bio-Rad, 152-8920), under o/n rocking at 22°. Assembled NDs were then purified via a Superdex-200 size exclusion column (GE Healthcare) and concentrated on a centrifugal concentrator [10 kDa molecular weight cutoff (MWCO), ThermoFisher Scientific]. The concentration of NDs was estimated by UV spectroscopy (*ε*^280^ = 18,450 M − 1 cm^−1^).

### NMR experiments

Experiments were performed using 50 μM ASAP1 PH. Samples (approx. 250 μL) were contained in Shigemi microcells. Data were acquired using Bruker AVIII-850 spectrometer equipped with a cryogenic TCI probe. All NMR data were processed and analyzed using Topspin 3.6.4 and/or NMRPipe^70^.

^1^H-^13^C HMQC spectra of ^13^CH_3_ methyl-labeled proteins were acquired using a SO-FAST HMQC pulse sequence as implemented in the NMRlib package^44^. The spectral widths were set to 12.94 and 25 ppm in the ^1^H and ^13^C dimensions, respectively, and inter-scan delays were set to 5 s. In total, 1542 × 256 complex points were recorded, and between 16 and 64 scans/FID gave rise to an acquisition time between 1.5 and 5 h. Prior to Fourier transformation, the data matrices were zero-filled to 4096 (^1^H) × 1024 (^13^C) complex points and multiplied by a cosine apodization function in both ^1^H and ^13^C dimensions. At EX conditions, ^1^H-^13^C HMQC spectra were acquired at 25 °C using the pulse sequence in the BRUKER pulse program library. Acquisition and processing parameters were the same as in the PB conditions.

At PB conditions, ^1^H-^15^N TROSY-HSQC spectra were acquired at 40 °C using the BEST TROSY principle as implemented in the NMRlib package^44^. The spectral widths were set to 12.06 and 36 ppm in the ^1^H and ^15^N dimensions, respectively, with a recycle delay set to 5 s. In total, 1542 × 256 complex points were recorded, and 96–128 scans/FID gave rise to an acquisition time between 6 and 8 h. Prior to Fourier transformation, the data matrices were zero-filled to 4096 (^1^H) × 1024 (^15^N) complex points and multiplied by a cosine apodization function in both dimensions. At EX conditions, ^1^H-^15^N TROSY-HSQC spectra were acquired at 25 °C using the trosyetf3gpsi pulse sequence in the BRUKER pulse program library. Acquisition and processing parameters were the same as in the PB conditions.

The PRE relaxation rate $\left({\varGamma }_{2,{i}}\right)$ on the observed proton from an unpaired electron on 5-doxyl PC was estimated as follows:

4 $${\varGamma }_{2,\,i}=\kappa \left(4{\tau }_{c}+\,\frac{3{\tau }_{c}}{1+{\omega }_{H}^{2}{\tau }_{c}^{2}}\right){r}^{-6}$$

where *κ* is 1.23 × 10^−32^ cm^6^ s^−2^ for the proton spin as reported previously^53^, *r* is the distance between the electron and nuclear spin for a single conformation of the protein at the surface, ${\tau }_{c}$ is the rotational correlation time of the electron–nuclear interaction, which was approximated using Eq. 5

5 $$\frac{1}{{\tau }_{c}}=\frac{1}{{\tau }_{R}}+\frac{1}{{\tau }_{S}}$$

where ${\tau }_{R}$ is protein rotational correlation time and ${\tau }_{S}$ is the electronic longitudinal relaxation time.

The error was calculated by the formula in Eq. 6

6 $${Error}=\frac{I}{I0\,}\,\sqrt{{{\left(\frac{1}{S/N}\right)}^{2}+\left(\frac{1}{S/N}\right)}_{0}^{2}}$$

where *I*, (*S/N*) and *I*_0_, (*S/N*)_0_ are the intensity and signal-noise ratios of resonance measured in paramagnetic and diamagnetic samples, respectively.

### Reporting summary

Further information on research design is available in the Nature Portfolio Reporting Summary linked to this article.

## Supplementary information

Transparent Peer Review File

Supplementary Information

Reporting Summary

## Data Availability

Additional data supporting this study are available within the Supplementary Information. Experimental data and scripts for paramagnetic relaxation enhancement calculations and fitting routines are available in the GitHub repository with the identifier https://github.com/criosx/nmr-pre-modeling. All other data that support the findings of this study are available from the corresponding authors upon reasonable request.
