# Supplementary material for: Binding orientation of weakly associating membrane peripheral proteins via membrane paramagnetic relaxation enhancement NMR
Source: Commun Chem. 2026 Apr 24;9:221. doi: 10.1038/s42004-026-02037-z (PMC13315575; doi:10.1038/s42004-026-02037-z)
Supplement: Supplementary file 2 — Supplementary Information [file 42004_2026_2037_MOESM2_ESM.pdf]

SUPPLEMENTARY INFORMATION

Binding orientation of weakly associating membrane peripheral proteins via membrane Paramagnetic Relaxation Enhancement NMR

Olivier Soubias, Frank Heinrich, Paul A. Randazzo and R. Andrew Byrd

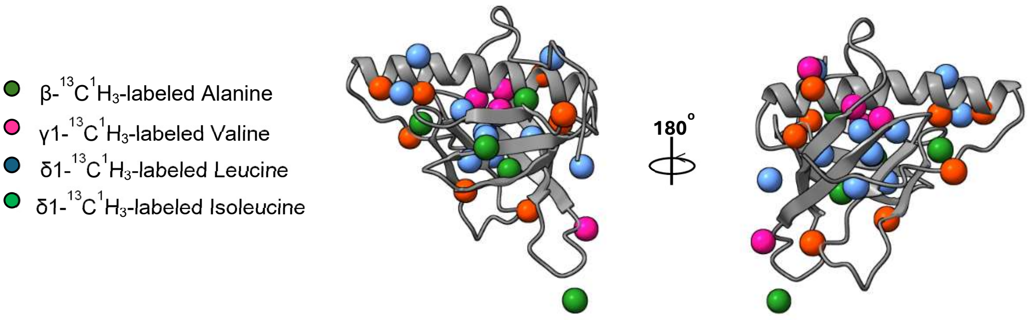

Supplementary Figure 1. Distribution of AILV methyl probes used as reporters in [325-451] ASAP1 PH domain.

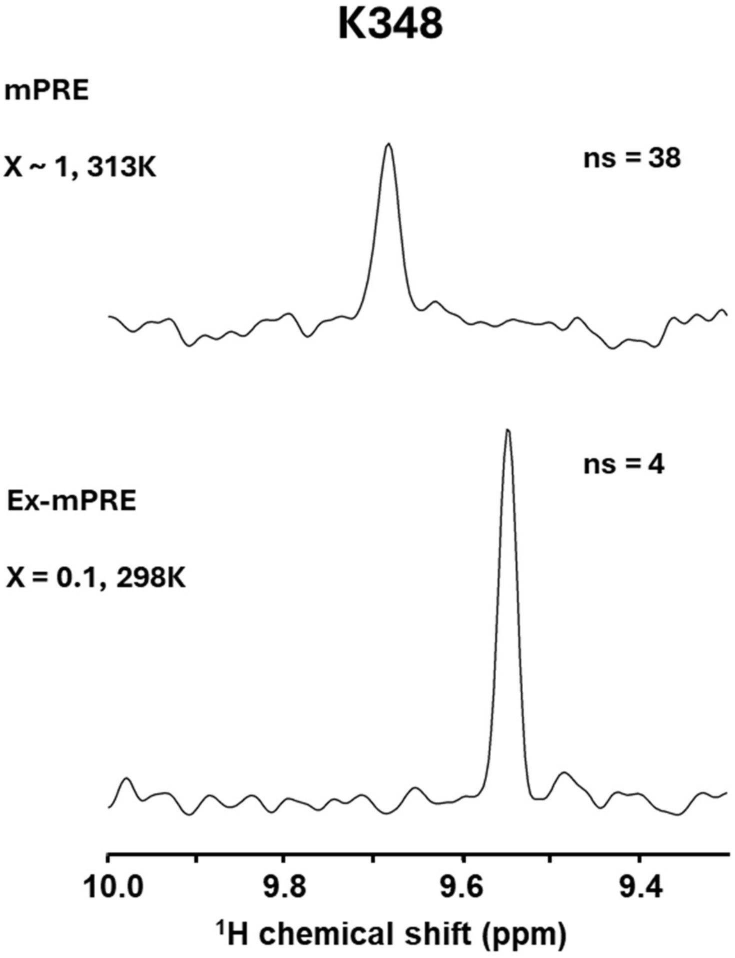

Supplementary Figure 2. One-dimensional ^{1}H traces corresponding to the F1 projection of the ^{1}H–^{15}N BEST-TROSY HSQC for residue K348 (U-^{2}H,^{15}N-labeled ASAP1 PH), shown for conditions with a bound fraction of $\chi \approx 1$ recorded at 313 K with 38 scans (top) and for a bound fraction of $\chi = 0.1$ recorded at 298 K with 4 scans (bottom)

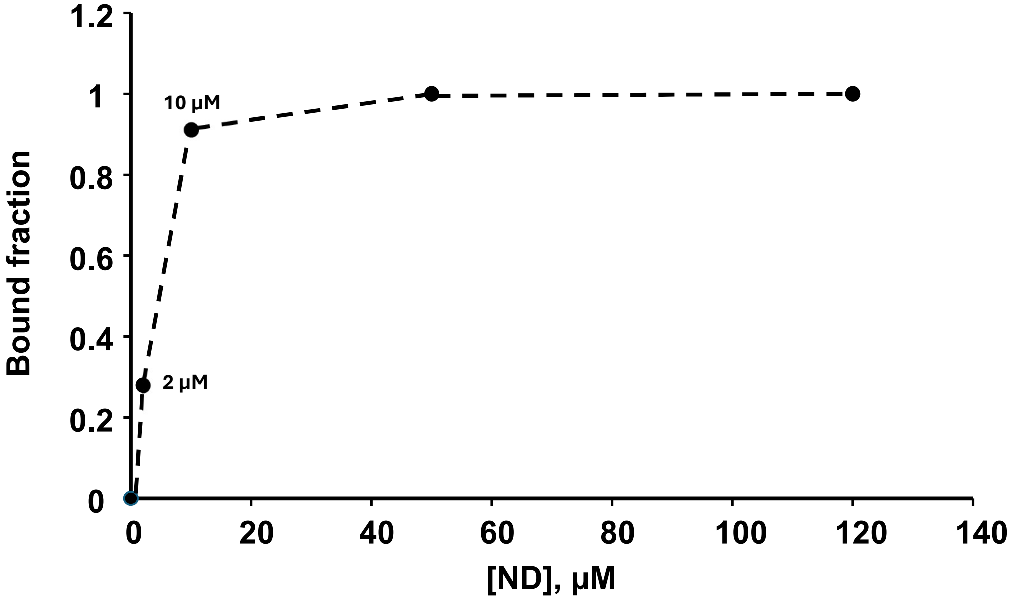

Supplementary Figure 3. Bound fraction of the PH domain, derived from the ^{1}H–^{13}C CSP of residue I371, plotted against the concentration of nanodiscs containing 15 mol% 18:1–18:1-PI(4,5)P_2. The dotted is solely provided to guide the eyes.

Parameter	PB conditions<br>5 mol% PIP	PB conditions<br>5 mol% PIP<br>H^N PRE only	PB conditions<br>5 mol% PIP<br>H^{CH3} PRE only
β [°]	21 ± 5	40 ± 20	30 ± 10
σ^β[°]	42 ± 4	30 ± 20	20 ± 10
γ [°]	67 ± 2	75 ± 8	70 ± 10
σ^γ [°]	21 ± 3	50 ± 40	30 ± 20
correlation	0.1 ± 0.4	0.0 ± 0.6	-0.6 ± 0.6
Distance d [Å]	29.5 ± 0.4	29.3 ± 0.5	28.6 ± 0.7
σ^d [Å]	2.2 ± 0.5	0.9 ± 0.8	1 ± 1
Goodness of fit, chi-squared	1.9(1)	1.5(1)	2.6(3)

Supplementary Table 1. Summary of the parameter values that model the NMR-PRE data obtained at PB conditions (5 mol% PIP) using a single MVN distribution of orientations. β and γ are the Euler angles defining a central orientation with standard deviations σ_β and σ_γ and a correlation ρ between the two angles. The distance of ASAP1 PH from the membrane is modeled separately as a normal distribution centered on a central distance (*d*) with standard deviation (σ^d).

Sample number	[5-Doxyl PC] (mol%)	χmbne
1	2	0.06
2	6	0.05
3	6	0.11
4	6	0.2
5	10	0.055
6	10	0.1
7	10	0.2

Supplementary Table 2. 5-Doxyl PC concentration and bound fraction (χ^{mbne}) of the samples used to measure mPRE at EX conditions in that study.

Parameter	EX conditions<br>5 mol% PIP	EX conditions<br>5 mol% PIP<br>H^N PRE only	EX conditions<br>5 mol% PIP<br>H^{CH3} PRE only
β [°]	19 ± 6	18 ± 6	30 ± 10
σ^β[°]	20 ± 10	30 ± 10	20 ± 10
γ [°]	70 ± 20	50 ± 20	100 ± 30
σ^γ [°]	40 ± 10	30 ± 10	90 ± 60
correlation	-0.1 ± 0.7	0.2 ± 0.7	-0.2 ± 0.7
Distance d [Å]	29 ± 1	29 ± 1	30 ± 2
σ^d [Å]	2 ± 1	3 ± 1	3 ± 1
Goodness of fit, chi-squared	1.5(1)	1.7(1)	0.7(4)

Supplementary Table 3. Summary of the parameter values that model the NMR-PRE data obtained at EX conditions (5 mol% PIP) using a single MVN distribution of orientations. β and γ are the Euler angles defining a central orientation with standard deviations σ_β and σ_γ and a correlation ρ between the two angles. The distance of ASAP1 PH from the membrane is modeled separately as a normal distribution centered on a central distance (*d*) with standard deviation (σ^d).

Parameter	PB conditions<br>15 mol% PIP	PB conditions<br>15 mol% PIP<br>H^N PRE only	PB conditions<br>15 mol% PIP<br>H^{CH3} PRE only
β [°]	30 ± 20	30 ± 10	40 ± 8
$σ^β$[°]	30 ± 20	50 ± 10	30 ± 20
γ [°]	130 ± 50	130 ± 50	80 ± 20
$σ^γ$ [°]	60 ± 30	70 ± 30	30 ± 20
correlation	0.0 ± 0.9	0.0 ± 0.7	-0.1 ± 0.5
Distance d [Å]	30.2 ± 0.8	30.4 ± 0.6	29 ± 1
$σ^d$ [Å]	3.2 ± 0.6	3.8 ± 0.6	3 ± 1
Goodness of fit, chi-squared	2.1(1)	2.1(1)	0.6(4)

Supplementary Table 4. Summary of the parameter values that model the NMR-PRE data obtained at PB conditions (15 mol% PIP) using a single MVN distribution of orientations. β and ɣ are the Euler angles defining a central orientation with standard deviations $σ_β$ and $σ_ɣ$ and a

correlation ρ between the two angles. The distance of ASAP1 PH from the membrane is modeled separately as a normal distribution centered on a central distance (d) with standard deviation (σ^d).
