# Supplementary material for: Binding orientation of weakly associating membrane peripheral proteins via membrane paramagnetic relaxation enhancement NMR
Source: Commun Chem. 2026 Apr 24;9:221. doi: 10.1038/s42004-026-02037-z (PMC13315575; doi:10.1038/s42004-026-02037-z)
Supplement: Supplementary file 1 — Transparent Peer Review File [file 42004_2026_2037_MOESM1_ESM.pdf]

nature portfolio

Peer Review File

Binding orientation of weakly associating membrane peripheral proteins via membrane Paramagnetic Relaxation Enhancement NMR

Corresponding Author: Dr Olivier soubias

This file contains all reviewer reports in order by version, followed by all author rebuttals in order by version.

Version 0:

Reviewer comments:

Reviewer #1

(Remarks to the Author)
Soubias et al. report a new NMR methodology, termed EX-mPRE, that allows to derive orientation of protein to membranes even in conditions where the protein main population is in the solvent, in fast exchange with the membrane-bound state. This builds on many previous PRE methodology publications and is a direct follow up of the authors' 2020 paper (10.1126/sciadv.abd1882) that already reported membrane PRE data, as well as neutron reflectometry and MD simulations.

Although the improvement from the 2020 paper is incremental, it is my opinion that the new EX-mPRE method has value in itself. Furthermore, it is particularly well validated given the wealth of previous data from the group. I therefore recommend publication and mostly have minor comments below. One major comment is that the modelling and fitting routine must be made accessible and documented so that analysis can be reproduced and other scientist can use it for other systems.

Minor comments:

• The nanodisc (lipid) concentration is estimated rather indirectly through monitoring selected protein chemical shift changes. I am surprised this would be the most accurate way. Can it not be monitored from lipid peak intensities, or calibrated from SEC profiles of nanodiscs? Please detail why this choice was made.

• I have not been able to find detailed protocols or code for the modelling, forward model (PRE calculation) and fitting routine. Is it accessible in a github repository? This must be made accessible and sufficiently documented.

• Equation 2. Was this derived or taken from the literature? Please detail and add a reference.

• The conclusion that the rate of exchange is fast compared to 1H-R2 is not supported. What is the rate of exchange? Was it measured or reported elsewhere?

• Similarly, later in the section about PIP2 clustering, the claim that PIP2 strengthens and slows exchange is not quantified and/or a reference is missing.

• Concentration-dependent experiment provides same information as varied g-factors in PRE tags. Some references would be nice to add here.

• Methods section: HMCQ should read HMQC

Reviewer #2

(Remarks to the Author)
The manuscript by Soubias and co-workers describes a method for using paramagnetic relaxation enhancement (PRE) NMR to determine how proteins associate to membranes. They use a model protein, the PIP-binding ASAP1 pleckstrin

homology domain, to investigate how the PRE method compares to previous results using neutron reflectometry. They optimese the method by varying temperature and water suppression methodology in order to maximize signal intensities for amide protons. They use 5-doxyl lipid to study the immersion of the protein in a nanodisc bilayer.

The paper is well-written and clearly presented. However, it is not obvious to this reviewer what the novel aspects of the methods are composed of. Recent examples of using similar or equivalent PRE methods to investigate the dynamic association of proteins to bilayers include Jin et al. JACS 146, 2023; Wu et al. JACS 148, 2026; for a review see for example Lenard et al. Progress in Nuclear Magnetic Resonance Spectroscopy, 132-133, 2022. Moreover, there are many different methods available to study membrane – protein interactions, for example various fluorescence methods such as FCS, FCCS and FRET. Solid-state NMR is also frequently used to study membrane-protein interactions. The authors could elaborate on why PRE NMR would be preferred over such methods and comment on the resolution and applicability for studies of transient membrane interactions.

The authors infer from their measurements that the regions in the protein that are mainly affected by the the 5-doxyl moiety is immersed in the bilayer (on what appears to be page 6, there are no page numbers in the manuscript). Have they considered how the doxyl-group behaves in the bilayer and where it is located? There are several studies available that have investigated the location of the spin-label in the bilayer by both PREs and fluorescence spectroscopy. This should be commented on. Since this is a methods paper, overall more comparisons to similar studies should be made.

On the same topic, the model in Figure 2G indicates that the protein is bound to the head-group region of the lipids. The authors claim (on page 7(?)) that the modelling data agree very well with PREs. It would be nice, however, to include some semi-quantitative data on how deep the residues are immersed in the bilayer. Using some “ruler” model they should be able to do so from their PRE data and compare to the model.

Again, on page 8 (?) it is stated that some residues “deeply penetrate the membrane”. They should clarify what they mean by this. There is a lack of (quantitative) data on details in how the protein is situated in the bilayer.

The authors should be careful using the word “membrane” when speaking of the nanodisc bilayer.

Reviewer #3

(Remarks to the Author)
Soubias and colleagues present a study establishing membrane PRE (mPRE) NMR and exchange-based EX-mPRE as an experimentally accessible approach to define membrane docking geometries of weakly associating peripheral proteins. Using the ASAP1 PH domain as a benchmark system, the authors convincingly reproduce the previously reported neutron reflectometry orientation while additionally revealing an orientational distribution in solution. The work is carefully executed, quantitatively analyzed, and clearly written.

The EX-mPRE strategy is especially compelling, as it extends PRE methodology into the fast-exchange regime and allows structural information to be extracted from low-population bound states . This will likely be broadly useful for the membrane protein structural biology community.

That said, several points deserve clarification or further discussion to further strengthen the present manuscript.

1. Many individual elements of the study have been described previously, also by the authors of the present manuscript. The authors should more clearly articulate what constitutes the principal novelty and what has not been possible before. Clarifying whether the advance lies in methodological integration, theoretical formalization, or biological application would sharpen the manuscript’s positioning. In particular a 2013 JACS paper by the Ikura group (dx.doi.org/10.1021/ja312508w) addresses a very similar problem with a tethered small GTPase, showing nucleotide-dependent changes in membrane orientation.

2. Small-angle X-ray scattering (SAXS) has also been used to study the shape of membrane-associated complexes. While SAXS lacks the strong distance weighting of PRE, it can provide low-resolution shape and orientation information in solution. Thus, a brief discussion comparing mPRE to SAXS would be valuable or using both methods in an integrated manner.

3. A previous study (https://doi.org/10.1002/chem.201800812) investigated membrane binding of full-length BclxL using PRE, RDCs, and MD. This is a relevant precedent demonstrating that orientation can be refined by combining multiple NMR observables. Even though in that study the relative orientation of the TMH and the peripheral domain was refined by HN-RDCs, RDC might still be a good tool to help quantify orientation changes of ASAP1-PH upon increasing PI(4,5)P_2 concentration. While technically more demanding, RDCs might complement PRE-derived distances and further constrain tilt and roll angles. A discussion of this possibility would strengthen the methodological framework.

4. The loss of backbone amide signals in predominantly bound conditions is attributed largely to enhanced transverse relaxation from membrane association. However, can conformational exchange on μs–ms timescales be excluded here?

5. The authors use 5-DOXYL-labeled lipids which places the paramagnetic center within the membrane interior. Is the

resulting rather constraint position of the nitroxide label crucial for obtaining accurate binding poses with the mPRE method? Or would Gd3+-headgroup-chelated lipids also suffice? A brief discussion of why 5-DOXYL was preferred would enhance methodological transparency.

6. In Figure 1D, an overlay of 2D-spectra acquired with and without band-selective pulses would visually strengthen the technical optimization claim in a better way than the 1D projections shown in Fig. 1A,B. Also, showing a comparison of 2D spectra used for the analysis of the mPRE versus the Ex-mPRE data would be valuable to demonstrate the potential advantage of the latter for larger systems.

7. The paper mentions that correct binding geometries can be obtained by MD alone. Thus, the question is how accurate the mPRE restraints really have to be. Once the membrane-binding interface is mapped by NMR, could extended, unbiased MD simulations alone converge to the correct docking orientation? A discussion of the complementary roles of experimental restraints versus MD sampling would clarify the added value of mPRE in systems where MD is already widely used.

8. The broadened orientational distribution at higher PIP2 concentrations is interpreted as expanded electrostatic sampling. However, a discussion on other, less specific reasons should be included, such as increased nonspecific electrostatic attraction or altered membrane surface charge density leading to reduced orientation specificity. Could the widened distribution reflect partial loss of specific binding geometry rather than enhanced dynamic sampling? And how about the biological role of such orientational changes? Is this a prerequisite for partner protein binding at the membrane? Maybe the authors can show a known complex structure and compare it to the binding pose obtained here. I would anticipate that the orienting effect of a partner protein is much more pronounced than changes in the lipid composition.

9. What is the anticipated molecular weight and membrane affinity ranges for the EX-mPRE method?

Version 1:

Reviewer comments:

Reviewer #1

(Remarks to the Author)
The authors have appropriately answered my comments. I have no further concerns.

Reviewer #2

(Remarks to the Author)
The manuscript has been significantly improved. The authors have added an introductory discussion on pros and cons with various available methods for examining membrane immersion.

Although the methodology described here offers a limited addition to methods previously described (by them) they have now better clarified what the novel aspects in the current paper are.

Reviewer #3

(Remarks to the Author)
In the revised version of this manuscript the authors have fully addressed all of my previous comments: the manuscript now clearly highlights its novelty, justifies experimental choices, provides additional methodological discussion (SAXS, RDCs, MD, EX-mPRE limits), and includes enhanced data presentation. As a result the manuscript has been strengthened significantly and now provides a very balanced discussion on the applicability of the Ex-mPRE method. My concerns have been fully resolved, and the manuscript is in my view now suitable for publication.

Open Access This Peer Review File is licensed under a Creative Commons Attribution 4.0 International License, which permits use, sharing, adaptation, distribution and reproduction in any medium or format, as long as you give appropriate credit to the original author(s) and the source, provide a link to the Creative Commons license, and indicate if changes were made.
In cases where reviewers are anonymous, credit should be given to 'Anonymous Referee' and the source.
The images or other third party material in this Peer Review File are included in the article's Creative Commons license, unless indicated otherwise in a credit line to the material. If material is not included in the article's Creative Commons license and your intended use is not permitted by statutory regulation or exceeds the permitted use, you will need to obtain permission directly from the copyright holder.
To view a copy of this license, visit https://creativecommons.org/licenses/by/4.0/

Reviewer #1 (Remarks to the Author):

Soubias et al. report a new NMR methodology, termed EX-mPRE, that allows to derive orientation of protein to membranes even in conditions where the protein main population is in the solvent, in fast exchange with the membrane-bound state. This builds on many previous PRE methodology publications and is a direct follow up of the authors' 2020 paper (10.1126/sciadv.abd1882) that already reported membrane PRE data, as well as neutron reflectometry and MD simulations.

Although the improvement from the 2020 paper is incremental, it is my opinion that the new EX-mPRE method has value in itself. Furthermore, it is particularly well validated given the wealth of previous data from the group. I therefore recommend publication and mostly have minor comments below. One major comment is that the modelling and fitting routine must be made accessible and documented so that analysis can be reproduced and other scientist can use it for other systems.

Minor comments:

• The nanodisc (lipid) concentration is estimated rather indirectly through monitoring selected protein chemical shift changes. I am surprised this would be the most accurate way. Can it not be monitored from lipid peak intensities, or calibrated from SEC profiles of nanodiscs? Please detail why this choice was made.

Response: We thank the reviewer for the opportunity to clarify our strategy. Specifically, small difference in ND concentration or small difference in PIP2 concentration in the nanodiscs can lead to variations in PH domain recruitment that exceed the margin of error necessary for mPRE experiments. Consequently, we determined that refining the concentration obtained initially by UV-Vis spectroscopy by monitoring the fraction of PH domain bound provided the most accurate measurement of the ND concentration. Regarding the suggestion to use lipid peak intensities, this approach was unfortunately not viable because the Doxyl group induces significant line-broadening and suppresses the intensity of nearby lipid proton signals. This interference makes ^{1}H NMR integration of the choline signal for instance unreliable for accurate lipid quantification.

The text was clarified.

• I have not been able to find detailed protocols or code for the modelling, forward model (PRE calculation) and fitting routine. Is it accessible in a github repository? This must be made accessible and sufficiently documented.

Response: In accordance with the reviewer's recommendation, we have now compiled the complete modeling protocols, the forward model scripts for Paramagnetic Relaxation Enhancement calculations, and the fitting routines into a dedicated GitHub repository. We have also updated the "Data Availability" of the manuscript to include the reference.

• Equation 2. Was this derived or taken from the literature? Please detail and add a reference.

Response: This equation was taken from established literature regarding the PRE-derived distance constraints in biomolecular complexes. We have now explicitly detailed the derivation context in the revised manuscript and added the appropriate reference to the text immediately following the equation.

• The conclusion that the rate of exchange is fast compared to 1H-R2 is not supported. What is the rate of exchange? Was it measured or reported elsewhere?

Response: We thank the reviewer for the opportunity to clarify the basis for assigning the exchange between free and membrane bound ASAP1 PH to the fast exchange regime. While we did not directly measure k_{ex} in the present study, quantitative estimates can be derived from previously reported binding parameters. In earlier work, we determined that ASAP1 PH binds to membranes containing 5 mol% PI(4,5)P_2 with a dissociation constant of K_d = 12 +/- 3 μM. Assuming a diffusion limited association rate (k_{on}), the corresponding dissociation rate (k_{off}) is expected to fall in the range of 10000 to 20000 s-1.

Even at the highest PRE values observed in this study, the relaxation decays remain well described by a single exponential. This behavior is consistent with exchange that is significantly faster than the PRE derived rates supporting the conclusion that the system resides in the fast exchange regime. Under these conditions, the measured PREs represent a true population weighted average of the free and membrane bound states.

We have added this quantitative justification to the revised manuscript.

• Similarly, later in the section about PIP2 clustering, the claim that PIP2 strengthens and slows exchange is not quantified and/or a reference is missing.

Response:

We thank the reviewer for this helpful comment. In the revised manuscript, we now clarify why higher PIP_2 density strengthens binding and shifts the system toward the intermediate-to-slow exchange regime. In our titrations, residue I371 reaches the chemical-shift position corresponding to the fully membrane-bound state at substantially lower total nanodisc concentration when using 15 mol% $PI(4,5)P_2$ (Figure SI3) compared to 5 mol% or 1 mol% (Soubias et al, Sci. Advances, 2020 (supplemental)). From these data, we estimate that the apparent affinity of the ASAP1 PH domain for nanodiscs containing 15 mol% PIP_2 is on the order of 1 μM or tighter. This supports the conclusion that increasing phosphoinositide density enhances the effective binding strength and slows exchange on the NMR timescale.

We also note in the revised text that the stoichiometry of binding under these conditions (low [ND], high [PIP]) is inherently difficult to determine, because at high PIP_2 density and low nanodisc concentration multiple PH domains may bind to a single nanodisc. This makes it challenging to extract a precise PH:ND ratio from chemical-shift changes alone, and the observed shift toward slow exchange likely reflects both stronger individual binding events and the increased probability of multivalent occupancy.

Finally, we have added references to established work (e.g., Knight and Falke, *Biophys. J.* 96, 2009) demonstrating that local clustering of multivalent lipids such as PIP_2 creates high-affinity binding patches that reduce the off-rate of peripheral membrane proteins. These studies provide a mechanistic framework consistent with our observations and support the interpretation that PIP_2 clustering strengthens membrane association and slows exchange.

• Concentration-dependent experiment provides same information as varied g-factors in PRE tags. Some references would be nice to add here.

Response: Thank you. The following references were added.

Clore, G.M. Practical Aspects of Paramagnetic Relaxation Enhancement in Biological Macromolecules. *Methods Enzymol* **564**, 485 97 (2015).

Iwahara, J., Schwieters, C.D. & Clore, G.M. Ensemble approach for NMR structure refinement against (1)H paramagnetic relaxation enhancement data arising from a flexible paramagnetic group attached to a macromolecule. *J Am Chem Soc* **126**, 5879-96 (2004).

• Methods section: HMCQ should read HMQC

Response: We thank the reviewer for carefully reading the manuscript. It was corrected in the revised version of the manuscript.

Reviewer #2 (Remarks to the Author):

The manuscript by Soubias and co-workers describes a method for using paramagnetic relaxation enhancement (PRE) NMR to determine how proteins associate to membranes. They use a model protein, the PIP-binding ASAP1 pleckstrin homology domain, to investigate how the PRE method compares to previous results using neutron reflectometry. They optimize the method by varying temperature and water suppression methodology in order to maximize signal intensities for amide protons. They use 5-doxyl lipid to study the immersion of the protein in a nanodisc bilayer.

The paper is well-written and clearly presented. However, it is not obvious to this reviewer what the novel aspects of the methods are composed of. Recent examples of using similar or equivalent PRE methods to investigate the dynamic association of proteins to bilayers include Jin et al. JACS 146, 2023; Wu et al. JACS 148, 2026; for a review see for example Lenard et al. Progress in Nuclear Magnetic Resonance Spectroscopy, 132-133, 2022. Moreover, there are many different methods available to study membrane – protein interactions, for example various fluorescence methods such as FCS, FCCS and FRET. Solid-state NMR is also frequently used to study membrane-protein interactions. The authors could elaborate on why PRE NMR would be preferred over such methods and comment on the resolution and applicability for studies of transient membrane interactions.

Response: We thank the reviewer for highlighting these elegant studies, which are now cited in the revised manuscript. In response, we expanded both the *Introduction* and the *Conclusion and Perspectives* to discuss the range of available approaches for characterizing membrane–protein and membrane–peptide interactions and emphasize how mPRE can provide powerful, residue-level information on the membrane orientation of weakly binding peripheral membrane proteins, and how these PRE-based NMR methods integrate naturally with complementary techniques such as MD simulations, neutron reflectometry, SANS, and NMR RDCs. This added discussion clarifies the unique strengths of PRE-based NMR for transient membrane systems while situating it within a broader, multi-method framework for defining membrane-binding geometry.

The authors infer from their measurements that the regions in the protein that are mainly affected by the the 5-doxyl moiety is immersed in the bilayer (on what appears to be page 6, there are no page numbers in the manuscript). Have they considered how the doxyl-group behaves in the bilayer and where it is located? There are several studies available that have investigated the location of the spin-label in the bilayer by both PREs and fluorescence spectroscopy. This should be commented on. Since this is a methods paper, overall more comparisons to similar studies should be made.

On the same topic, the model in Figure 2G indicates that the protein is bound to the head-group region of the lipids. The authors claim (on page 7(?)) that the modelling data agree very well with PREs. It would be nice, however, to include some semi-quantitative data on how deep the residues are immersed in the bilayer. Using some "ruler" model they should be able to do so from their PRE data and compare to the model.

Again, on page 8 (?) it is stated that some residues "deeply penetrate the membrane". They should clarify what they mean by this. There is a lack of (quantitative) data on details in how the protein is situated in the bilayer.

Response: We thank the reviewer for these constructive comments. We agree that it is important to more explicitly address the behavior and positioning of the 5-doxyl spin label within the bilayer, as well as to provide a clearer, semi-quantitative description of the protein's immersion depth.

Although we did not directly measure the depth distribution of the 5-doxyl group in the present work, extensive MD simulations, EPR and fluorescence studies have shown that the 5-doxyl nitroxide adopts a well-defined Gaussian distribution centered approximately 8 ± 3 Å

below the phosphate plane of the bilayer. We have now incorporated these references into the manuscript and clarified why 5-doxyl PC is an appropriate probe for mPRE measurements. We also note that, due to the large magnetic moment of the nitroxide electron, PRE effects remain detectable for nucleus–electron distances up to ~25 Å, enabling us to extract meaningful distance information from the observed attenuation profiles.

To address the reviewer's request for semi-quantitative depth information, we have added a new panel (Fig. 2B) and accompanying text that maps the measured PRE-derived (I/I_0) values onto approximate distances from the spin label. For example, the (I/I_0) ≈ 0.4 observed for residues K350 and W357 corresponds to an average distance of ~18 Å from the nitroxide, placing the tip of the β_1/β_2 loop approximately 3 Å beneath the phosphate headgroup plane. This analysis clarifies what we mean by "deep penetration" and provides a quantitative framework for interpreting the immersion of specific residues and to guide the reader.

The authors should be careful using the word "membrane" when speaking of the nanodisc bilayer.

Response: We agree that while the nanodisc contains a phospholipid bilayer, it possesses distinct physical constraints, such as lateral pressure profiles and the presence of the membrane scaffold protein boundary, that, among other parameters, distinguish it from an expansive, continuous biological membrane. In the revised manuscript, we have replaced reserved the term "*membrane*" for broad biological contexts and replaced it otherwise with "*nanodisc bilayer*" or "*lipid bilayer* ".

Reviewer #3 (Remarks to the Author):

Soubias and colleagues present a study establishing membrane PRE (mPRE) NMR and exchange-based EX-mPRE as an experimentally accessible approach to define membrane docking geometries of weakly associating peripheral proteins. Using the ASAP1 PH domain as a benchmark system, the authors convincingly reproduce the previously reported neutron reflectometry orientation while additionally revealing an orientational distribution in solution. The work is carefully executed, quantitatively analyzed, and clearly written.

The EX-mPRE strategy is especially compelling, as it extends PRE methodology into the fast-exchange regime and allows structural information to be extracted from low-population bound states . This will likely be broadly useful for the membrane protein

structural biology community.

That said, several points deserve clarification or further discussion to further strengthen the present manuscript.

1. Many individual elements of the study have been described previously, also by the authors of the present manuscript. The authors should more clearly articulate what constitutes the principal novelty and what has not been possible before. Clarifying whether the advance lies in methodological integration, theoretical formalization, or biological application would sharpen the manuscript's positioning. In particular a 2013 JACS paper by the Ikura group (dx.doi.org/10.1021/ja312508w) addresses a very similar problem with a tethered small GTPase, showing nucleotide-dependent changes in membrane orientation.

Response: We thank the reviewer for raising this important point. We have clarified in the revised manuscript that the principal novelty of our work lies in extending mPRE to *non-tethered, conditionally membrane-bound peripheral membrane proteins*, a class of systems that differ fundamentally from the tethered small GTPase examined in the 2013 JACS study. In tethered systems, the membrane-bound state is effectively enforced, yielding a high local concentration that is readily accessible to PRE-based analysis. By contrast, the proteins studied here are only weakly associated with membranes. The methodological advances presented here, including optimized acquisition strategies for conventional mPRE and the development of the EX-mPRE approach, specifically address this challenge by enabling extraction of membrane-orientation information from low-affinity, dynamically exchanging systems that could not be studied using prior PRE-based methods.

To make this distinction explicit, we added the following text to the Introduction: "Importantly, unlike earlier PRE-based studies on tethered membrane proteins such as small GTPases, the systems examined here belong to the class of non-tethered, conditionally membrane-bound peripheral proteins whose weak, reversible, and lipid-dependent interactions present a different biophysical challenge."

2. Small-angle X-ray scattering (SAXS) has also been used to study the shape of membrane-associated complexes. While SAXS lacks the strong distance weighting of PRE, it can provide low-resolution shape and orientation information in solution. Thus, a brief discussion comparing mPRE to SAXS would be valuable or using both methods in an integrated manner.

Response: We appreciate the reviewer's suggestion to discuss the role of Small-angle X-ray scattering (SAXS) in the context of our study. We agree that a multimodal approach is essential for a comprehensive understanding of membrane-associated complexes and have emphasized that message in the "conclusion and perspectives" paragraph.

While SAXS is an excellent tool for determining the global envelope of macromolecular complexes in solution, it faces significant challenges when applied to peripheral membrane proteins associated with nanodiscs. The primary difficulty involves accurate signal subtraction and contrast. The scattering profile of a protein-nanodisc complex is a composite of the protein, the scaffold, and the lipid bilayer. Because the electron density of the lipid tails is similar to the buffer, while the headgroups are significantly denser, the background is non-uniform. Furthermore, protein binding can induce subtle structural changes in the bilayer itself, such as alterations in thickness or area per lipid, which makes subtraction of the "empty" nanodisc often imperfect. Consequently, SAXS typically provides a low-resolution shape that may obscure the precise protein-lipid interface. Small-angle Neutron Scattering (SANS) and Neutron Reflectometry (NR) partially address these contrast issues through solvent deuteration and contrast variation. NR, in particular, provides exceptional precision in measuring the profile of the protein mass along the membrane normal. We have integrated these points into the "Conclusion and Perspectives" paragraph to highlight how mPRE provides not only standalone but also essential, complementary information to scattering techniques and other methods.

3. A previous study (https://doi.org/10.1002/chem.201800812) investigated membrane binding of full-length BclxL using PRE, RDCs, and MD. This is a relevant precedent demonstrating that orientation can be refined by combining multiple NMR observables. Even though in that study the relative orientation of the TMH and the peripheral domain was refined by HN-RDCs, RDC might still be a good tool to help quantify orientation changes of ASAP1-PH upon increasing PI(4,5)P_2 concentration. While technically more demanding, RDCs might complement PRE-derived distances and further constrain tilt and roll angles. A discussion of this possibility would strengthen the methodological framework.

Response: We thank the reviewer for reminding us of the elegant study on Bcl-xL, which serves as an excellent precedent for the power of combining multiple NMR observables to define the orientation of protein at the membrane surface. We agree that incorporating RDCs would provide powerful constraints on the tilt and roll angles of the ASAP1-PH domain, complementing the distance-based restraints provided by mPRE.

Applying RDCs to highly charged peripheral membrane proteins such as ASAP1-PH presents several significant challenges. The first arises from the interaction between the protein and the alignment medium. Most commonly used alignment media, including Pf1 bacteriophage and many negatively charged liquid-crystalline phases, rely on electrostatic interactions to generate a stable alignment tensor. Many peripheral membrane proteins, however, are themselves highly charged and depend on both specific and nonspecific electrostatic interactions for membrane recruitment. ASAP1-PH, for example, is strongly basic and binds membranes through a delicate electrostatic balance. Under these conditions, the alignment medium is likely to compete directly with the nanodisc for protein binding as it is observed for ASAP1-PH in the presence of Pf1. Neutral alignment media such as stretched polyacrylamide gels offer an alternative, but they often lack stability in the presence of lipid assemblies or restrict the rotational diffusion required to obtain high-quality spectra of large complexes.

Overcoming these hurdles would be highly impactful. One possible direction would be to orient the nanodisc directly without relying on external alignment media such that the nanodisc would turn into a self-aligning reference frame, enabling simultaneous acquisition of RDCs and mPREs under native binding conditions.

We have added a discussion of these possibilities to the revised manuscript, emphasizing that although mPRE currently provides the most direct and robust measure of membrane immersion, integrating RDCs through self-aligning nanodiscs represents a powerful next step toward a more complete description of peripheral membrane protein orientation at the bilayer surface.

4. The loss of backbone amide signals in predominantly bound conditions is attributed largely to enhanced transverse relaxation from membrane association. However, can conformational exchange on μs–ms timescales be excluded here?

Response: We thank the reviewer for raising this important point. In the revised manuscript, we clarify the basis for our interpretation of the missing backbone amide signals under predominantly bound conditions. We hypothesized that the widespread loss of amide cross peaks arises primarily from two global effects: (1) attenuation of amide $^{1}\mathrm{H}_{\mathrm{N}}$ intensities due to increased water exchange and spin diffusion in the highly viscous

membrane-containing samples, and (2) rapid transverse relaxation associated with the slow overall tumbling of the >100 kDa protein–nanodisc complex.

We agree that localized μs–ms conformational exchange within flexible loops may contribute to additional line broadening. However, the near-uniform disappearance of signals across the structured core of the protein argues against conformational exchange as the dominant mechanism. Instead, the global nature of the attenuation is more consistent with the combined effects of water exchange and slow molecular motion, which are expected to impact most residues irrespective of local dynamics.

We have revised the text to explicitly acknowledge that μs–ms exchange cannot be fully excluded at specific sites, while emphasizing that the overall pattern of signal loss is best explained by these global relaxation mechanisms.

5. The authors use 5-DOXYL-labeled lipids which places the paramagnetic center within the membrane interior. Is the resulting rather constraint position of the nitroxide label crucial for obtaining accurate binding poses with the mPRE method? Or would Gd3+-headgroup-chelated lipids also suffice? A brief discussion of why 5-DOXYL was preferred would enhance methodological transparency.

Response: We thank the reviewer for raising this important point. We chose 5-Doxyl-PC over headgroup-chelated paramagnetic probes, such as Gd3+-headgroup-chelated lipids, to preserve the native electrostatic environment of the membrane-water interface. Highly charged surface probes interfere with the basic residues of the ASAP1-PH domain, potentially perturbing the PI(4,5)P_2-dependent binding mode. By sequestering the nitroxide moiety within the hydrophobic core at the C-5 position, we minimize these interfacial artifacts. The choice of an acyl-chain-embedded label is further supported by MD simulations, which indicate that headgroup-conjugated labels, such as Tempo-PC, exhibit complex partitioning behavior and concentration-dependent depth fluctuations. In contrast, the immersion depth of the 5-Doxyl group is strictly governed by the acyl chain conformation, showing negligible deviation from the position of the corresponding unlabeled carbon atom. We have revised the text accordingly.

6. In Figure 1D, an overlay of 2D-spectra acquired with and without band-selective pulses would visually strengthen the technical optimization claim in a better way than the 1D projections shown in Fig. 1A,B. Also, showing a comparison of 2D spectra used for the

analysis of the mPRE versus the Ex-mPRE data would be valuable to demonstrate the potential advantage of the latter for larger systems.

Response: In the revised manuscript, Figure 1 has been updated to include these comparisons. Specifically, Figure 1A now features a side by side comparison of the 2D spectra acquired with and without band-selective pulses, illustrating sensitivity gain. We have retained the 1D projections alongside these 2D spectra.

Furthermore, to address the comparison between mPRE and Ex-mPRE data, we have included 2D spectra for both methods. This comparison highlights the superior performance of the Ex-mPRE approach for larger systems, where signal overlap and relaxation properties will limit HN mPRE for complexes > 150 kDa.

We believe these additions significantly strengthen the methodological validation presented in the paper.

7. The paper mentions that correct binding geometries can be obtained by MD alone. Thus, the question is how accurate the mPRE restraints really have to be. Once the membrane-binding interface is mapped by NMR, could extended, unbiased MD simulations alone converge to the correct docking orientation? A discussion of the complementary roles of experimental restraints versus MD sampling would clarify the added value of mPRE in systems where MD is already widely used.

Response: We thank the reviewer for this question regarding the synergy between mPRE restraints and MD simulations. While unbiased MD simulations have made significant strides in predicting peripheral membrane binding, they often face two major hurdles: the extensive timescales required for rotational convergence of the protein on the bilayer and the sensitivity of the binding pose to the chosen lipid force fields.

Rather than viewing them as redundant, we see mPRE and MD as deeply complementary. In our workflow, the NMR data may serve to prune the conformational space, allowing the MD simulations to refine the side-chain interactions within a physically validated "global" orientation. This prevents the simulation from becoming trapped in metastable, non-native orientations that are common in purely computational docking. We have added a dedicated paragraph to the Conclusion and perspectives paragraph section to clarify this relationship.

8. The broadened orientational distribution at higher PIP2 concentrations is interpreted as expanded electrostatic sampling. However, a discussion on other, less specific reasons should be included, such as increased nonspecific electrostatic attraction or altered membrane surface charge density leading to reduced orientation specificity. Could the widened distribution reflect partial loss of specific binding geometry rather than enhanced dynamic sampling? And how about the biological role of such orientational changes? Is this a prerequisite for partner protein binding at the membrane? Maybe the authors can show a known complex structure and compare it to the binding pose obtained here. I would anticipate that the orienting effect of a partner protein is much more pronounced than changes in the lipid composition.

Response: We thank the reviewer for this insightful comment. We agree that the broadened orientational distribution at higher PIP_2 concentrations may reflect a combination of factors, including not only expanded opportunities for specific $PI(4,5)P_2$–sidechain contacts but also nonspecific interactions. We have revised the text to explicitly acknowledge these additional mechanisms and to clarify that the observed broadening may represent both enhanced dynamic sampling and a partial relaxation of specific binding geometry.

Regarding the biological implications, we now discuss how lipid-dependent modulation of orientational freedom could influence the regulation of multidomain membrane binding domains-containing proteins, particularly in the context of partner-protein binding at the membrane. Although we do not include a structural comparison to a known complex in this work, we note that the mPRE method is well positioned to address such questions in future studies by examining how lipid composition and partner interactions may both determine membrane-associated orientations. We have incorporated this expanded discussion into the revised manuscript.

9. What is the anticipated molecular weight and membrane affinity ranges for the EX-mPRE method?

Response: We thank the reviewer for this question. The EX-mPRE approach is designed for systems that reside in the fast-to-intermediate exchange regime on the NMR timescale,

which typically corresponds to membrane affinities in the low tens of micromolar to millimolar range. This affinity window ensures that the exchange between free and membrane-bound states remains sufficiently rapid for PREs to report on population-weighted averages. With respect to molecular size, the anticipated upper limit for amide-based EX-mPRE is comparable to that of classical solution-state TROSY NMR of globular complexes, on the order of ~150 kDa. This limit can be extended somewhat through the use of 3D experiments to alleviate spectral overlap. If the experiment is performed using only side-chain methyl probes, the accessible molecular-weight range increases substantially. Methyl-TROSY detection remains effective for particles in the 300–500 kDa range, and in favorable cases even larger assemblies can be studied. Thus, a methyl-based EX-mPRE implementation would allow the method to be applied to significantly larger membrane-associated complexes while preserving sensitivity to exchange-dependent PRE effects. This is now discussed in the final paragraph.

REVIEWERS' COMMENTS:

Reviewer #1 (Remarks to the Author):

The authors have appropriately answered my comments. I have no further concerns.

Response: We thank the reviewer for critically reading the manuscript and for their thoughtful comments, which helped improve the clarity and overall quality of the work.

Reviewer #2 (Remarks to the Author):

The manuscript has been significantly improved. The authors have added an introductory discussion on pros and cons with various available methods for examining membrane immersion.

Although the methodology described here offers a limited addition to methods previously described (by them) they have now better clarified what the novel aspects in the current paper are.

Response: We thank the reviewer for their careful evaluation and constructive feedback, which contributed to strengthening the manuscript.

Reviewer #3 (Remarks to the Author):

In the revised version of this manuscript the authors have fully addressed all of my previous comments: the manuscript now clearly highlights its novelty, justifies experimental choices, provides additional methodological discussion (SAXS, RDCs, MD, EX-mPRE limits), and includes enhanced data presentation. As a result the manuscript has been strengthened significantly and now provides a very balanced discussion on the applicability of the Ex-mPRE method. My concerns have been fully resolved, and the manuscript is in my view now suitable for publication.

Response: We thank the reviewer for their detailed review and valuable suggestions that enhanced the presentation, rigor and scope of the manuscript.
